# Evidence for histamine release in chronic inducible urticaria – A systematic review

**DOI:** 10.3389/fimmu.2022.901851

**Published:** 2022-07-28

**Authors:** Kanokvalai Kulthanan, Martin K. Church, Eva Maria Grekowitz, Tomasz Hawro, Lea Alice Kiefer, Kanyalak Munprom, Yanisorn Nanchaipruek, Chuda Rujitharanawong, Dorothea Terhorst-Molawi, Marcus Maurer

**Affiliations:** ^1^ Department of Dermatology, Faculty of Medicine Siriraj Hospital, Mahidol University, Bangkok, Thailand; ^2^ Institute of Allergology, Charité – Universitätsmedizin Berlin, Corporate Member of Freie Universität Berlin and Humboldt-Universität zu Berlin, Berlin, Germany; ^3^ Fraunhofer Institute for Translational Medicine and Pharmacology ITMP, Allergology and Immunology, Berlin, Germany; ^4^ Department of Dermatology, Allergology and Venereology, Institute and Comprehensive Center for Inflammation Medicine, University Medical Center Schleswig-Holstein, Lübeck, Germany

**Keywords:** antihistamines, chronic inducible urticaria, histamine, mast cell, wheal

## Abstract

**Background:**

Chronic inducible urticaria (CIndU) constitutes a group of nine different CIndUs in which pruritic wheals and/or angioedema occur after exposure to specific and definite triggers. Histamine released from activated and degranulating skin mast cells is held to play a key role in the pathogenesis of CIndU, but evidence to support this has, as of yet, not been reviewed systematically or in detail. We aim to characterize the role and relevance of histamine in CIndU.

**Methods:**

We systematically searched 3 electronic databases (PubMed, Scopus, and Embase) for studies that reported increased serum or skin histamine concentration (direct evidence) or *in vitro* or *ex vivo* histamine release (indirect evidence) following trigger exposure.

**Results:**

An initial total of 3,882 articles was narrowed down to 107 relevant studies of which 52 were in cold urticaria, 19 in cholinergic urticaria, 14 in heat urticaria, 10 in contact urticaria, 7 each in solar urticaria and vibratory angioedema, 4 each in symptomatic dermographism and aquagenic urticaria, and 3 in delayed pressure urticaria. The results of our review support that histamine has a key pathogenic role in the pathogenesis of all CIndUs, but it is not the sole mediator as evidenced by the often poor relationship between the level of histamine and severity of symptoms and the variable clinical efficacy of H_1_-antihistamines.

**Conclusions:**

Histamine released from skin mast cells is a key driver of the development of signs and symptoms and a promising therapeutic target in CIndU.

## Introduction

Chronic inducible urticaria (CIndU) is a subgroup of chronic urticaria in which recurrent pruritic wheals and/or angioedema occur after exposure to specific and definite triggers (1). There are nine different CIndUs, with a wide spectrum of physical and chemical triggers. Examples of the former are exposure to cold and pressure in cold urticaria (ColdU) and delayed pressure urticaria (DPU), respectively. Examples of the latter are water in aquagenic urticaria (AquaU) and sweat in cholinergic urticaria (CholU). How these triggers cause the occurrence of wheals, angioedema, or both, in patients with CIndUs, is largely unclear (2, 3).

Histamine, released from activated and degranulating skin mast cells, is held to play a key role in the pathogenesis of CIndU. This is supported by several independent lines of evidence. First, the wheals that occur in patients with CIndU share many features of those induced by histamine skin prick testing or intracutaneous injection (4–6). Both occur within minutes of exposure, and they are, in most cases, pruritic and short lived, with resolution after minutes to a few hours (4, 5). Second, immunoglobulin E (IgE) autoantibodies appear to be involved, in some CIndUs, in the activation of skin mast cells (1, 7–10). IgE-mediated activation is a well characterized mechanism of mast cell histamine release and relevant in allergies and chronic spontaneous urticaria (9). Third, H_1_-antihistamines (AH_1_) protect many, but not all, patients with CIndU from the occurrence of signs and symptoms (11–15). In addition, there is also direct evidence, from *in vivo* studies, as well as indirect evidence, from *in vitro* studies, for histamine release from skin mast cells in CIndU, but this evidence has, as of yet, not been reviewed systematically or in detail.

Here, we review both *in vivo* and *in vitro* evidence for histamine release as a pathogenic driver in CIndU. Our aim is to better characterize the role and relevance of histamine, and, by proxy, those of its receptors and its only relevant source in human skin, mast cells, as mechanisms of the development of signs and symptoms and relevant treatment targets in CIndU.

## Materials and methods

Preferred Reporting Items for Systematic Reviews and Meta-Analysis were used in this study (16). We systematically searched 3 electronic databases (PubMed, Scopus, and Embase) for studies that reported direct and/or indirect evidence of histamine release in CIndU and were published before April 2021 (inclusion criteria). Increased serum or skin histamine concentration in patients with CIndU after provocation testing was defined as direct evidence (17), whereas other evidence for histamine release was classified as indirect (18). The advanced search option was used with the Mesh terms “histamine” AND each type of CIndU, for example, “cold urticaria” or “symptomatic dermographism”. All articles identified were screened by at least two independent reviewers, and their reference lists were searched for additional reports of relevance. Case reports, case series, randomized controlled trials (RCTs), cohort, case-control, and cross-sectional studies that reported evidence of histamine release in CIndU were included. Owing to the relatively limited number of studies of histamine release in CIndU, case reports and case series were included, aiming to collect data from available published evidence as much as possible.

**Figure 1:**
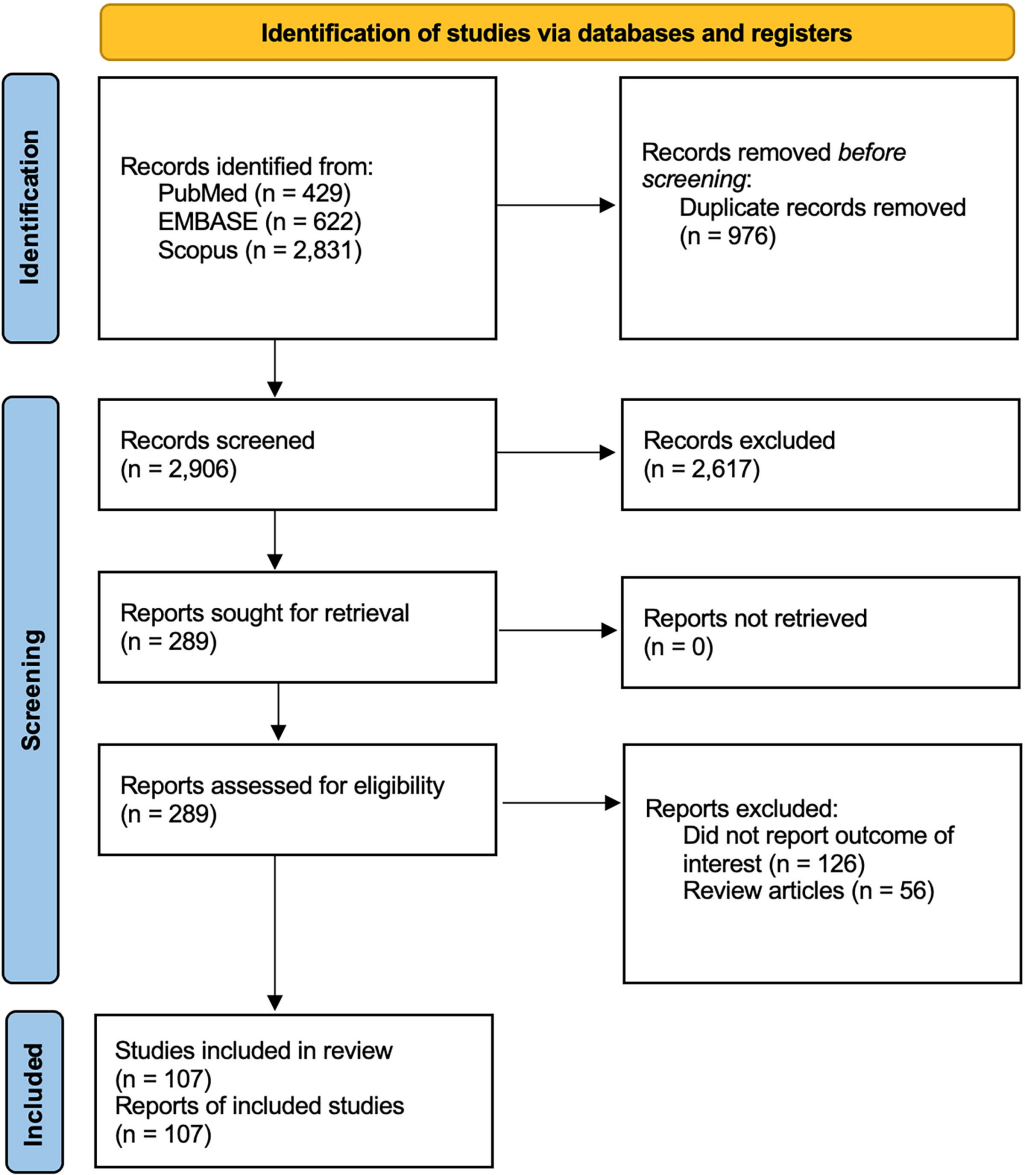
Flow diagram of the literature review in this systematic review. One hundred and seven articles met the inclusion criteria and were included in this systematic review (1 randomized controlled trial, 10 cohort studies, 9 case-control studies, 35 cross-sectional studies, 6 case series, and 46 case reports). There were 4 articles with SD, 52 articles with ColdU, 3 articles with DPU, 7 articles with SolU, 19 articles with CholU, 14 articles with HeatU, 7 articles with VA, 4 articles with AquaU, and 10 articles with ConU. Some of articles reported multiple chronic inducible urticaria cases. AquaU, aquagenic urticaria; CholU, cholinergic urticaria; ColdU, cold urticaria; ConU, contact urticaria; DPU, delayed pressure urticaria; HeatU, heat urticaria; SD, symptomatic dermographism; SolU, solar urticaria; VA, vibratory angioedema.

From each report included in our review, we extracted the number of studied patients, how histamine release was assessed and what the outcome was, as well as the author information and year of publication. Our search identified 3,882 potentially relevant articles (429 from PubMed, 622 from Embase, and 2,831 from Scopus). After the exclusion of 976 duplicates, 2,906 studies were reviewed by title and abstract. Of these, 2617 articles were excluded because they did not fulfill our inclusion criteria. A total of 289 articles underwent full-text review. In the end, 107 studies, consisting of 1 RCT, 10 cohort studies, 9 case-control studies, 35 cross-sectional studies, 6 case series and 46 case reports, were included in our systematic review (**Figure 1**). Of these 107 studies, 52 were in ColdU, 19 in CholU, 14 in heat urticaria (HeatU), 10 in contact urticaria (ConU), 7 each in solar urticaria (SolU) and vibratory angioedema (VA), 4 each in symptomatic dermographism (SD) and AquaU, and 3 in DPU. Some articles reported multiple types of CIndU. All articles were in English. The quality and risk of bias assessment of included articles in systematic review was determined (**Supplementary Table 1**).

The rating system developed by de Croon et al. (19) was applied to categorized the levels of evidence for ‘association’ and ‘no association’ The Levels of evidence for “association” were categorized as *strong*: 3 studies available that find an association in the same direction or ≥ 4 studies available, of which > 66% find a significant association in the same direction and no more than 25% find an opposite association, *weak*: 2 studies available that find a significant association in the same direction or 3 studies available, of which two find a significant association in the same direction and the third study finds no significant association, *no evidence*: ≤ 1 study available, *inconsistent*: remaining cases. The levels of evidence for “no association” were defined as *strong*: > 4 studies are available, of which >85% find no significant association, *weak*: > four studies are available, of which >75% find no significant association.

## Results

### Symptomatic dermographism 

Two studies provided direct evidence of an increase in blood histamine levels following provocation testing in symptomatic dermographism (SD). In the first, post-provocation blood histamine levels were increased in five of ten patients of whom two had markedly higher levels (20) (**Supplementary Table 2**). In the second, a patient with severe SD showed a marked increase in venous histamine from 18 ng/ml before scratching to a peak of 62 ng/ml two minutes after provocation. Blood histamine levels returned to baseline within 4 minutes (21).

In 1970, Greaves and Sondegaard reported direct evidence for histamine release in SD obtained by the use of fine bore needles to sample the skin in the provocation area of 8 patients and 16 controls (17). Histamine was detected in the basal perfusate of all 8 patients, probably due to whealing in response to needle insertion. None of the controls showed detectable basal histamine. In six of the 8 patients, stroking the skin produced further whealing accompanied by a further rise in skin histamine levels.

As for indirect evidence of histamine release in SD, a systematic review of RCTs and non-RCTs of treatment of SD revealed that first-generation H_1_-antihistamines (fgAH_1_) had variable efficacy and significant side effects, whereas second-generation H_1_-antihistamines (sgAH_1_), in all studies, were effective with a good safety profile and should be the first-line treatment (22).

### Cold urticaria 

That histamine is released in symptomatic patients with cold urticaria (ColdU) was first suggested by Bram Rose in 1941 who detected elevated blood histamine levels in these patients after cold challenge (20, 23) (**Supplementary Table 3**). This finding was confirmed by several subsequent studies, including the ones by Dunér et al. (24, 25), who found that blood histamine levels can also be elevated in healthy subjects after cold challenge, Spuzic *et al. (* 26), who found plasma histamine to be elevated in 3 of 7 ColdU patients washed with cold water, and others (27–30).

Perhaps the most definitive experiments were performed by Kaplan et al. (18, 31), who showed, in six ColdU patients, that immersion of one hand in cold water caused a rise in plasma histamine of 10-36 ng/ml, peaking at 4 minutes. The results of these reports on blood histamine levels are supported by studies in which histamine skin levels were found to be increased in cold-induced wheals (3). Using skin microdialysis, Andersson et al. (32) showed ice cube-induced increases in skin histamine levels varying from 91 to 550 nM in three patients. Nuutinen et al. (33) reported that of six patients, two had high dialysate histamine levels (621 and 1,269 nM) while the others had lower levels (21 – 100 nM). Kring Tannert et al. (10) also detected, by skin microdialysis, histamine release following cold challenge, which was much reduced after tolerance induction.

In a study with the sgAH_1_ bilastine, post-provocation skin microdialysis was performed in 20 ColdU patients on three occasions, following treatment for one week each with placebo, bilastine 20, and bilastine 80 mg (12). While only group mean data were reported in the published paper, results for individual patients are presented here.

**Figure 2:**
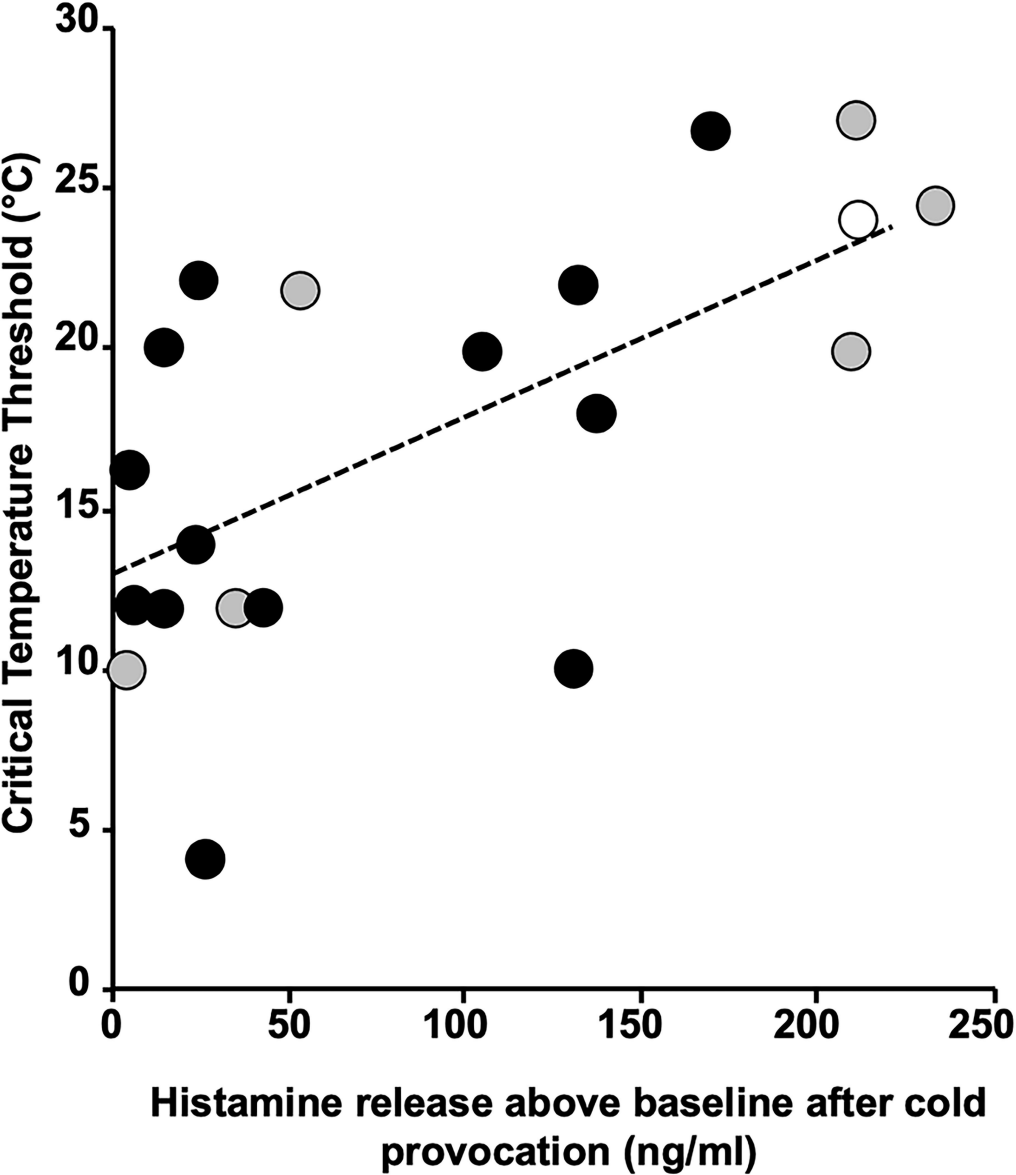
Regression plot of critical threshold temperatures against dialysate histamine concentrations. Patients are colour coded as to their responsiveness to 80 mg bilastine, green > 80% inhibition (responders), yellow 30–80% inhibition (partial responders) and red < 30% (nonresponders). The regression line is statistically significant (P = 0.003) and intercepts the critical threshold temperature axis at 13°C.

The results of microdialysis for each patient for the three occasions were remarkably consistent for each showing that the antihistamine, while significantly reducing the critical temperature threshold (CTT), had no effect on histamine release. Several conclusions may be made from the results shown in **Figure 2**. First, the increases in histamine release at the temperature at which wheals occurred were very variable, from 3 – 222 nM. Second, there is a significant (*P* = 0.003) relationship between dialysate histamine concentrations and CTTs suggesting that histamine is a major mediator. Third, the regression line intercepts the CTT line at 13°C indicating strongly that there is participation of another mediator in addition to histamine in producing the symptoms. Fourth, the patients with the highest histamine levels are in general less sensitive to 80 mg bilastine, the antihistamine used in this study.

Studies that provided indirect evidence for histamine release in ColdU include those of Juhlin et al. (34), who reported that mast cells in biopsies of ColdU patients show signs of degranulation, and of Kaplan et al. (35), who demonstrated histamine release by *ex vivo* cooling and rewarming of skin biopsies from ColdU patients. One interesting feature of these studies is the wide variability of histamine release, ranging from 14 to 128 ng/ml (3).

Sigler et al. (36) showed that the fgAH_1_ cyproheptadine can prevent cold-induced hives, and a recent systematic review by Dressler et al. showed that sgAH_1_ were more effective than placebo in the treatment of patients with ColdU. Standard and high dose treatment were more effective than placebo in terms of the rates of patients who became “symptom free” (37).

### Delayed pressure Urticaria 

Direct evidence of histamine release in DPU comes from 3 studies. Two of them measured histamine release in skin lesions of DPU patients. Kaplan et al. (3) assessed suction blister fluid obtained at 2-hour intervals from skin sites exposed to pressure and found histamine levels to be increased between 4-10 hours, with peak levels of approximately 30 ng/ml at 6-8 hours (**Supplementary Table 4**). Czarnetzki et al. (38) reported that mean histamine levels were elevated in wheals induced by pressure. They also reported indirect evidence that peripheral blood leukocytes had significantly increased intracellular histamine levels and increased histamine releasability.

Mijailović et al. (39) reported that pressure challenge on a patient’s forearm caused increased histamine levels in blood collected from the draining cubital vein 14 hours later as compared with the contralateral cubital vein and to baseline. Of note, 18 hours after challenge the patient developed systemic anaphylaxis and received adrenaline.

For indirect evidence, a systematic review of treatment of DPU showed that sgAH_1_ were effective in 3 RCTs. Combining a sgAH_1_ with montelukast (2 RCTs) or theophylline (1 non-RCT) was more effective than the sgAH_1_ alone. There are no studies on sgAH_1_ updosing in DPU (40).

### Solar urticaria 

Direct evidence for histamine release in solar urticaria (SolU) comes mostly from blood histamine assessment: Two patients with confirmed SolU were challenged and histamine was measured by Soter et al. (41) (**Supplementary Table 5**) Serum histamine rose from baseline of <0.1 ng/ml to peak levels of 7.0 and 37 ng/ml at 5 minutes. Levels returned to baseline by 20 minutes.

Hawk et al. studied histamine release in four SolU patients provoked by UVB (42). The results showed that two patients had increased histamine levels of 20 ng/ml and 8.5 ng/ml at 2 and 5 minutes after irradiation, respectively while the other two showed smaller increases. Additionally, electron microscopy of skin specimens taken after challenge showed numerous mast cells undergoing exocytotic changes, characteristically seen during histamine release, whereas in some cells no signs of degranulation were found.

Keahey et al. (6) measured venous blood histamine levels in three patients before and after UVA irradiation with various intervals up to 30 min compared with after induction of tolerance by repeated exposure to UVA. The results showed small and variable histamine release, which was reduced by tolerance induction. Watanabe et al. (43) reported increase in venous histamine in two SolU patients when wheals developed approximated 30 minutes after UV irradiation.

There is only one study, of Kaplan et al. (3), that performed histamine measurements in the skin. SolU was induced in one patient by ultraviolet (UV) light filtered through window glass for 30 seconds. Histamine levels in suction blisters induced over urticarial lesions and the contralateral non-urticarial skin were 127 ng/ml and 24 ng/ml, respectively.

As for indirect evidence, Baart de Faille et al. (44) reported that histological evaluation revealed higher numbers of mast cells in irradiated specimens, as well as appearance of degranulation 24 hours after irradiation, implying histamine release. Also, AH_1_ treatment is of benefit in 35-75% of SolU patients (45–47).

### Heat-induced urticaria 

Histamine release in heat-induced urticaria **(**HeatU) was demonstrated by 14 studies, all of which have adopted an *in vivo* approach and showed a homogeneous picture with histamine rising to peak levels in venous blood draining the exposed site within minutes of exposure (14, 20, 48–57) (**Supplementary Table 6**). Most studies chose exposure temperatures of 40°C, going up to 45°C, with one exception of 56°C due to the high individual threshold of the examined patient (49). These results from venous blood are also supported by a study in which suction blister histamine peaked at 30 minutes after heat challenge, whereas it did not rise in healthy controls (15). Most of the blood studies did not have healthy controls who underwent the same procedures, but those who did showed no rise of histamine levels upon exposure to heat (50). Two studies reported that after successful treatment of HeatU patients with a desensitization protocol, patients remained symptom free and the previously seen rise of histamine could no longer be detected upon exposure to heat (50, 56).

As for indirect evidence, one study examined skin biopsies after heat provocation using electron microscopy, revealing features of mast cell degranulation (58). Also, approximately 60% of patients with HeatU respond to sgAH_1_, although only few patients achieve complete control (59).

### Vibratory angioedema 

Vibratory angioedema (VA) is divided into 2 subgroups, hereditary vibratory angioedema (HVA) and acquired vibratory angioedema (AVA), with histamine release being purported to be relevant for both subgroups.

Direct evidence of histamine release in HVA is reported in 3 studies. Metzger et al. (60) and Kaplan et al. (31) studied the same patient whose forearm was applied on a vortex mixer for 4 minutes on different occasions **Supplementary Table 7**). Venous histamine levels peaked at 22 and 53 ng/ml at 1-3 minutes and declined to baseline at 4-13 minutes. A similar study was reported by Boyden et al. (61), with serum histamine levels peaking at 90 and 130 nmol/l within 5 minutes after challenge in two patients.

Four *in vivo* investigations of histamine release provide direct evidence in AVA, using blood obtained before and after vibratory stimulation for 1-5 min at various intervals. Ting *et al. (* 62) reported peak venous blood histamine of 24 ng/ml at 1 minute. Wener et al. (63) detected peak histamine of 18 ng/ml at 5 minutes. Keahey et al. (5) reported 2 patients having peak levels of serum histamine of 1,224 to 9,000 pg/ml at 5 minutes and a secondary rise at 3-4 hours. Zhao et al. (64) reported mean peak serum histamine levels of 9 ng/ml in 3 patients at 30 minutes after stimulation.

As for indirect evidence, three studies demonstrated mast cells in various stages of degranulation at provocation sites, as assessed by immunohistochemistry or electron microscopy (5, 61, 62). Also, VA patients can benefit from AH_1_ treatment (65).

### Cholinergic urticaria 

The first report of direct evidence of histamine release in three patients with cholinergic urticaria (CholU) was by Kaplan et al. in 1975 (18) (**Supplementary Table 8**). Baseline venous blood histamine levels were ≤1 ng/ml. The first patient had mild symptoms and a peak histamine level of 3 ng/ml during exercise, which returned to baseline within 20 minutes. The second patient had more severe symptoms and a peak histamine level of 25 ng/ml during exercise, which was still elevated (4 ng/ml) at 35 minutes. The third patient had no itch accompanying the skin lesions and showed no detectable histamine release after exercise challenge. Variable elevations of venous blood histamine with exercise were reported in other studies (11, 14, 29, 31, 66–70).

Indirect evidence of histamine release was demonstrated in five recent *in vitro* studies including 84 patients using basophil histamine release tests (71–76). Although sgAH_1_ are used as first-line treatment for patients with CholU, many patients report only mild to moderate improvement. The addition of AH_2_ was effective in some patients with refractory cases of CholU (77). A systematic review by Dressler et al. showed that fgAH_1_ and sgAH_1_ are more effective than placebo, with increased doses resulting in higher efficacy compared with placebo (37).

### Contact urticaria

Contact urticaria (ConU) may be divided into immunologic and non-immunologic ConU. Among 11 studies reporting direct evidence of histamine release in immunologic ConU, 20 cases were provoked by latex (78–80), and 1 each by limonium tataricum (81), cereal flour extract (82), rice (83), polyvinylpyrrolidone (84), and chlorhexidine (85) **Supplementary Table 9**). These urticariogenic substances all released histamine from patient leucocytes *in vitro* (78–83).

For *in vivo* results, serum histamine levels were assessed in 2 cases. The histamine levels of the patient with chlorhexidine allergy were measured before and after skin prick test and patch test. The results showed an increase of more than 3-fold and 2-fold after skin prick test and patch test, respectively (85).

In an *in vivo* study of nettle induced non-immunologic contact urticaria, histamine levels rose to 35 ± 240 nM at 15 minutes after challenge and declined rapidly (86).

For indirect evidence of ConU, sgAH_1_ are effective in controlling both the number and the duration of wheals in most patients with ConU had a good response and relieved the symptoms. In uncontrolled cases, updosing of antihistamines can be helpful (87).

### Aquagenic urticaria 

*In vivo* histamine release in AquaU was demonstrated in two studies (67, 88) (**Supplementary Table 10**). Following skin provocation testing, increased plasma histamine levels were reported in 2 patients. Davis et al. reported peak plasma histamine of 2 ng/ml at 60 minutes after challenge, which returned to baseline within 2 hours (67). Sibbald et al. reported plasma histamine increase of up to 11 ng/ml after challenge (88). Acetone enhanced the plasma histamine increase in response to subsequent challenge, but it did not evoke wheals by itself (88).

As for indirect evidence, two studies demonstrated mast cell degranulation at sites of provocation in one patient each (88, 89). Also, an *in vitro* study of basophils from patients with AquaU, showed histamine release in response to challenge with all of 4 dilutions of human callus extract (90). FgAH_1_ and sgAH_1_ lead to benefit in most patients (91–93), and up-dosing can improve the response (94, 95).

## Discussion

Our systematic review demonstrates direct and indirect evidence of histamine release in all types of CIndUs and confirms the role of histamine in their pathogenesis. (**Supplementary Tables 11, 12**).

Comparisons of blood histamine levels before and after challenge was the most common *in vivo* approach and demonstrate the extent and kinetics of skin histamine release. The histamine detection methods used in these studies were various and varied over time. In earlier studies, use of guinea-pig ileum in a superfusion cascade system was frequently used (96, 97). In later studies more sensitive chemical methods were employed, including radioenzyme techniques (98–100) and competitive enzyme immunoassay (57, 101). Skin microdialysis was another *in vivo* method commonly used. Its advantages include that it does not require repeated blood sampling to assess the release of histamine in human skin (102).

Regarding *in vitro* methods, basophil tests were commonly performed to detect histamine release, e.g. by fluorometric assay (103–105), radioenzyme technique (98), enzymatic double-isotopic assay (106), enzyme-linked immunosorbent assay (107), and HPLC (72). Furthermore, demonstration of mast cell degranulation in skin biopsy by some studies provided indirect evidence that histamine might be involved in the development of signs and symptoms of CIndU. Because different studies used different methods with different sensitivity, the levels of histamine released are difficult to compare.

Although the exact pathogenesis of CIndU is still largely unclear (1), mast cell activation and degranulation with subsequent release of proinflammatory mediators are held to be critical and histamine is a main mediator. Other mediators and cytokines with pathogenic relevance in CIndU include tumor necrotic factor-α (108, 109), prostaglandin D_2_ (110–112), leukotriene (113), acetylcholine (114), platelet activating factor and interleukins (109, 115). However, the role of mediators other than histamine in each CIndU subtype remains ill defined, and their relevance should be explored in future studies. Treatment with AH_1_ in each CIndU can lead to various clinical responses ranging from non-response to complete response (12, 116). (Supplementary Table 11) Thus, it is likely that histamine is not of the same relevance in all CIndUs or in all patients with the same CIndU. Importantly, non-response to an AH_1_ does not rule out that histamine is a major CIndU driver, as histamine can also induce skin inflammation and itch *via* H_4_ receptors. H_4_ receptor antagonists should be assessed for their efficacy in the treatment of patients with CIndU (117). Also, further studies should explore and better define the spectrum and roles of mast cell mediators other than histamine that are involved in the induction of signs and symptoms of each CIndU. Finally, novel targeted therapies are needed and should be developed to improve the management of CIndUs (**Supplementary Table 13**).

## Conclusions

This review supports a key pathogenic role of histamine in all types of CIndUs. However, some points are still unclear, for example, the trigger pathway for histamine release and the relationship between the level of histamine and severity of symptoms.

Our systematic review identified direct and indirect evidence of histamine release by *in vivo* and/or *in vitro* analyses in all types of CIndUs. This should prompt further studies on the role of histamine receptors other than H1R, especially H4R in the pathogenesis of CIndUs.

## Data availability statement

The original contributions presented in the study are included in the article/**Supplementary Material**. Further inquiries can be directed to the corresponding author.

## Author contributions

KK, MC, and MM contributed to conception and design of the study. KK, KM, and CR built the research strategy. MC, EG, TH, LK, KM, YN, CR, and DT-M performed data extraction. MC, EG, TH, LK, KM, YN, CR, and DT-M wrote the first draft of the manuscript. KK, MC, and MM reviewed the manuscript. All authors contributed to manuscript revision, read, and approved the submitted version.

## Conflict of interest

The authors declare that the research was conducted in the absence of any commercial or financial relationships that could be construed as a potential conflict of interest.

## Publisher’s note

All claims expressed in this article are solely those of the authors and do not necessarily represent those of their affiliated organizations, or those of the publisher, the editors and the reviewers. Any product that may be evaluated in this article, or claim that may be made by its manufacturer, is not guaranteed or endorsed by the publisher.
