# Supplementary material for: Evidence for histamine release in chronic inducible urticaria – A systematic review
Source: Front Immunol. 2022 Jul 28;13:901851. doi: 10.3389/fimmu.2022.901851 (PMC9365951; doi:10.3389/fimmu.2022.901851)
Supplement: Supplementary file 1 [file Table_1.docx]

**Supplementary Table 1** Quality and risk of bias assessment of included articles in systematic review

| A. Randomized controlled trials | | | | | | |
| --- | --- | --- | --- | --- | --- | --- |
| **Study, year (Ref)** | **Random sequence generation**  **(selection bias)** | **Allocation concealment** | **Blinding of participants and personnel** | **Blinding of outcome assessment** | **Incomplete outcome data** | **Selective reporting** |
| Krause et al., 2013 (12) | + | + | + | + | + | + |

+, Low risk of bias

| B. Non-randomized controlled trials | | | | | | | | | | | | | | | |  |
| --- | --- | --- | --- | --- | --- | --- | --- | --- | --- | --- | --- | --- | --- | --- | --- | --- |
| Study, year (Ref) | **Criteria** | | | | | | | | **Additional criteria in the case of comparative study** | | | | | | | |
|  | **A stated aim of the study** | **Inclusion of consecutive patients** | **Prospective collection of data** | **End point appropriate to the study aim** | **Unbiased evaluation of end points** | **Follow-up period appropriate** | **Loss to follow-up not exceeding 5%** | **Prospective calculation of the study size** | | **A control group having the criterion standard intervention** | **Contemporary groups** | **Baseline equivalence of groups** | **Prospective calculation of the sample size** | **Statistical analyses adapted to the study design** | **Total** | |
| Rose et al., 1941 (20) | 2 | 2 | 2 | 2 | 0 | 2 | 2 | 0 | | - | - | - | - | - | 12 | |
| Greaves et al., 1970 (17) | 2 | 2 | 2 | 2 | 0 | 2 | 2 | 0 | | - | - | - | - | - | 12 | |
| Garafalo et al., 1981 (21) | 2 | 2 | 2 | 2 | 0 | 2 | 2 | 0 | | - | - | - | - | - | 12 | |
| Lawlor et al., 1989 (120) | 2 | 2 | 2 | 2 | 2 | 2 | 2 | 0 | | - | - | - | - | - | 14 | |
| Henderson et al., 1958 (121) | 2 | 2 | 2 | 2 | 0 | 2 | 2 | 0 | | - | - | - | - | - | 12 | |
| Dunér et al., 1960 (25) | 2 | 2 | 2 | 2 | 0 | 2 | 2 | 0 | | - | - | - | - | - | 12 | |
| Juhlin et al., 1961 (34) | 2 | 2 | 2 | 2 | 0 | 2 | 2 | 0 | | - | - | - | - | - | 12 | |
| Spuzic et al., 1961 (26) | 2 | 2 | 2 | 2 | 0 | 2 | 2 | 0 | | - | - | - | - | - | 12 | |
| Beall et al., 1963 (123) | 2 | 2 | 2 | 2 | 0 | 2 | 2 | 0 | | - | - | - | - | - | 12 | |
| Granerus et al., 1969 (124) | 2 | 2 | 2 | 2 | 0 | 2 | 2 | 0 | | - | - | - | - | - | 12 | |
| Kaplan et al., 1975 (18) | 2 | 2 | 2 | 2 | 0 | 2 | 2 | 0 | | - | - | - | - | - | 12 | |
| Kaplan et al., 1976 (31) | 2 | 2 | 2 | 2 | 0 | 2 | 2 | 0 | | - | - | - | - | - | 12 | |
| Soter et al., 1976 (27) | 2 | 2 | 2 | 2 | 0 | 2 | 2 | 0 | | - | - | - | - | - | 12 | |
| Bentley-Phillips et al., 1976 (28) | 2 | 2 | 2 | 2 | 0 | 2 | 2 | 0 | | - | - | - | - | - | 12 | |
| Bentley-Phillips et al., 1978 (125) | 2 | 2 | 2 | 2 | 1 | 2 | 2 | 0 | | - | - | - | - | - | 13 | |
| Kaplan et al., 1978 (3) | 2 | 2 | 2 | 2 | 0 | 2 | 2 | 0 | | - | - | - | - | - | 12 | |
| Black et al., 1980 (30) | 2 | 2 | 2 | 2 | 0 | 2 | 2 | 0 | | - | - | - | - | - | 12 | |
| Black et al., 1979 (126) | 2 | 2 | 2 | 2 | 0 | 2 | 1 | 0 | | - | - | - | - | - | 11 | |
| Sigler et al., 1979 (29) | 2 | 2 | 2 | 2 | 0 | 2 | 2 | 0 | | - | - | - | - | - | 12 | |
| Inoue et al., 1980 (127) | 1 | 2 | 2 | 2 | 0 | 2 | 2 | 0 | | - | - | - | - | - | 11 | |
| Keahey et al., 1980 (129) | 2 | 2 | 2 | 2 | 0 | 2 | 2 | 0 | | - | - | - | - | - | 12 | |
| Sigler et al., 1980 (36) | 2 | 2 | 2 | 2 | 0 | 2 | 2 | 0 | | - | - | - | - | - | 12 | |
| Akiyama et al., 1981 (130) | 2 | 2 | 2 | 2 | 0 | 2 | 2 | 0 | | - | - | - | - | - | 12 | |
| Black et al., 1981 (131) | 2 | 2 | 2 | 2 | 0 | 2 | 2 | 0 | | - | - | - | - | - | 12 | |
| Kaplan et al., 1981 (35) | 2 | 2 | 2 | 2 | 0 | 2 | 2 | 0 | | - | - | - | - | - | 12 | |
| Kaplan et al., 1981 (132) | 2 | 2 | 2 | 2 | 0 | 2 | 2 | 0 | | - | - | - | - | - | 12 | |
| Johnston et al., 1982 (133) | 1 | 2 | 2 | 2 | 0 | 2 | 2 | 0 | | - | - | - | - | - | 11 | |
| B. Non-randomized controlled trials | | | | | | | | | | | | | | | |  |
| Study, year (Ref) | **Criteria** | | | | | | | | **Additional criteria in the case of comparative study** | | | | | | | |
|  | **A stated aim of the study** | **Inclusion of consecutive patients** | **Prospective collection of data** | **End point appropriate to the study aim** | **Unbiased evaluation of end points** | **Follow-up period appropriate** | **Loss to follow-up not exceeding 5%** | **Prospective calculation of the study size** | | **A control group having the criterion standard intervention** | **Contemporary groups** | **Baseline equivalence of groups** | **Prospective calculation of the sample size** | **Statistical analyses adapted to the study design** | **Total** | |
| Wasserman et al., 1982 (134) | 2 | 2 | 2 | 2 | 0 | 2 | 2 | 0 | | - | - | - | - | - | 12 | |
| Katayama et al., 1983 (135) | 2 | 2 | 2 | 2 | 0 | 2 | 2 | 0 | | - | - | - | - | - | 12 | |
| Kaplan et al., 1984 (137) | 2 | 2 | 2 | 2 | 0 | 2 | 2 | 0 | | - | - | - | - | - | 12 | |
| Neittaanmäki et al., 1984 (138) | 2 | 2 | 2 | 2 | 0 | 2 | 2 | 0 | | 2 | 2 | 1 | 0 | 2 | 19 | |
| Wasserman et al., 1984 (139) | 1 | 2 | 2 | 2 | 0 | 2 | 2 | 0 | | 2 | 2 | 1 | 0 | 0 | 16 | |
| Grandel et al., 1985 (112) | 2 | 2 | 2 | 2 | 0 | 2 | 2 | 0 | | 2 | 2 | 1 | 0 | 0 | 17 | |
| Farnam et al., 1986 (48) | 1 | 2 | 2 | 2 | 0 | 2 | 2 | 0 | | - | - | - | - | - | 11 | |
| Heavey et al., 1986 (111) | 2 | 2 | 2 | 2 | 0 | 2 | 2 | 0 | | - | - | - | - | - | 12 | |
| Gruber et al., 1988 (8) | 2 | 2 | 2 | 2 | 0 | 2 | 2 | 0 | | 2 | 2 | 2 | 0 | 0 | 18 | |
| Keahey et al., 1988 (142) | 2 | 2 | 2 | 2 | 0 | 2 | 2 | 0 | | - | - | - | - | - | 12 | |
| Ormerod et al., 1988 (110) | 2 | 2 | 2 | 2 | 0 | 2 | 2 | 0 | | 2 | 2 | 2 | 0 | 2 | 20 | |
| Maltby et al., 1989 (113) | 2 | 2 | 2 | 2 | 0 | 2 | 2 | 0 | | - | - | - | - | - | 12 | |
| McClean et al., 1989 (14) | 1 | 2 | 2 | 2 | 0 | 2 | 2 | 0 | | - | - | - | - | - | 11 | |
| Anfosso-Capra et al., 1990 (143) | 1 | 2 | 2 | 2 | 0 | 2 | 2 | 0 | | - | - | - | - | - | 11 | |
| Orfan et al., 1991(144) | 2 | 2 | 2 | 2 | 0 | 2 | 2 | 0 | | - | - | - | - | - | 12 | |
| Miller et al., 1992 (145) | 2 | 2 | 2 | 2 | 0 | 2 | 2 | 0 | | - | - | - | - | - | 12 | |
| Rosenkranz et al., 1992 (146) | 2 | 2 | 2 | 2 | 0 | 2 | 2 | 0 | | 2 | 2 | 1 | 0 | 2 | 19 | |
| Tillie-Leblond et al., 1994 (108) | 2 | 2 | 2 | 2 | 0 | 2 | 2 | 0 | | - | - | - | - | - | 12 | |
| Andersson et al., 1995 (32) | 2 | 2 | 2 | 2 | 0 | 2 | 2 | 0 | | - | - | - | - | - | 12 | |
| Capulong et al., 1997 (147) | 2 | 2 | 2 | 2 | 0 | 2 | 2 | 0 | | - | - | - | - | - | 12 | |
| Asero et al., 2002 (148) | 1 | 2 | 2 | 2 | 0 | 2 | 0 | 0 | | - | - | - | - | - | 9 | |
| Nuutinen et al., 2007 (33) | 2 | 2 | 2 | 2 | 0 | 2 | 2 | 0 | | - | - | - | - | - | 12 | |
| Ota et al., 2010 (149) | 1 | 2 | 2 | 2 | 0 | 2 | 2 | 0 | | - | - | - | - | - | 11 | |
| Kring Tannert et al., 2012 (10) | 2 | 2 | 2 | 2 | 0 | 2 | 2 | 0 | | - | - | - | - | - | 12 | |
| Ellis et al., 2013 (150) | 2 | 2 | 2 | 2 | 0 | 2 | 2 | 0 | | - | - | - | - | - | 12 | |
| Meyer et al., 2013 (101) | 2 | 2 | 2 | 2 | 0 | 2 | 2 | 0 | | - | - | - | - | - | 12 | |
| Gorczyza et al., 2019 (151) | 2 | 2 | 2 | 2 | 2 | 2 | 0 | 0 | | - | - | - | - | - | 12 | |
| Czarnetzki et al., 1984 (38) | 2 | 2 | 2 | 2 | 0 | 2 | 0 | 0 | | 1 | 2 | 0 | 0 | 2 | 15 | |
| Mijailović et al., 1997 (39) | 2 | 2 | 2 | 2 | 0 | 2 | 2 | 0 | | - | - | - | - | - | 12 | |
| Soter et al., 1979 (41) | 2 | 2 | 2 | 2 | 0 | 2 | 2 | 0 | | - | - | - | - | - | 12 | |
| Baart de la Faille et al., 1975 (44) | 1 | 2 | 2 | 2 | 0 | 2 | 2 | 0 | | - | - | - | - | - | 11 | |
| Hawk et al., 1980 (42) | 2 | 2 | 2 | 2 | 0 | 2 | 2 | 0 | | - | - | - | - | - | 12 | |
| Keahey et al., 1984 (6) | 2 | 2 | 2 | 2 | 0 | 2 | 2 | 0 | | - | - | - | - | - | 12 | |
| Neittaanmäki et al., 1989 (153) | 1 | 2 | 2 | 2 | 0 | 2 | 2 | 0 | | - | - | - | - | - | 11 | |
| Watanabe et al., 1999 (43) | 2 | 2 | 2 | 2 | 0 | 2 | 2 | 0 | | - | - | - | - | - | 12 | |
| Atkins et al., 1981 (51) | 2 | 2 | 2 | 2 | 0 | 2 | 2 | 0 | | - | - | - | - | - | 12 | |
| Grant et al., 1981 (52) | 2 | 2 | 2 | 2 | 0 | 2 | 2 | 0 | | - | - | - | - | - | 12 | |
| B. Non-randomized controlled trials | | | | | | | | | | | | | | | |  |
| Study, year (Ref) | **Criteria** | | | | | | | | **Additional criteria in the case of comparative study** | | | | | | | |
|  | **A stated aim of the study** | **Inclusion of consecutive patients** | **Prospective collection of data** | **End point appropriate to the study aim** | **Unbiased evaluation of end points** | **Follow-up period appropriate** | **Loss to follow-up not exceeding 5%** | **Prospective calculation of the study size** | | **A control group having the criterion standard intervention** | **Contemporary groups** | **Baseline equivalence of groups** | **Prospective calculation of the sample size** | **Statistical analyses adapted to the study design** | **Total** | |
| Irwin et al., 1985 (49) | 2 | 2 | 2 | 2 | 0 | 2 | 2 | 0 | | - | - | - | - | - | 12 | |
| Koro et al., 1986 (53) | 2 | 2 | 2 | 2 | 0 | 2 | 2 | 0 | | - | - | - | - | - | 12 | |
| Neittaanmäki et al., 1988 (15) | 2 | 2 | 2 | 2 | 0 | 2 | 2 | 0 | | - | - | - | - | - | 12 | |
| Higgins et al., 1991 (54) | 2 | 2 | 2 | 2 | 0 | 2 | 2 | 0 | | - | - | - | - | - | 12 | |
| Baba et al., 1998 (50) | 2 | 2 | 2 | 2 | 0 | 2 | 2 | 0 | | - | - | - | - | - | 12 | |
| Skrebova et al., 2001 (55) | 2 | 2 | 2 | 2 | 0 | 2 | 2 | 0 | | - | - | - | - | - | 12 | |
| Fukunaga et al., 2002 (56) | 2 | 2 | 2 | 2 | 0 | 2 | 2 | 0 | | - | - | - | - | - | 12 | |
| Koh et al., 2002 (58) | 2 | 2 | 2 | 2 | 0 | 2 | 2 | 0 | | - | - | - | - | - | 12 | |
| Martín-Muñoz et al., 2002 (57) | 2 | 2 | 2 | 2 | 0 | 2 | 2 | 0 | | - | - | - | - | - | 12 | |
| Metzger et al., 1976 (60) | 2 | 2 | 2 | 2 | 0 | 2 | 2 | 0 | | - | - | - | - | - | 12 | |
| Boyden et al., 2016 (61) | 2 | 2 | 2 | 2 | 0 | 2 | 2 | 0 | | - | - | - | - | - | 12 | |
| Ting et al., 1983 (62) | 2 | 2 | 2 | 2 | 0 | 2 | 2 | 0 | | - | - | - | - | - | 12 | |
| Wener et al., 1983 (63) | 2 | 2 | 2 | 2 | 0 | 2 | 2 | 0 | | - | - | - | - | - | 12 | |
| Keahey et al., 1987 (5) | 2 | 2 | 2 | 2 | 0 | 2 | 2 | 0 | | - | - | - | - | - | 12 | |
| Zhao et al., 2019 (64) | 2 | 2 | 2 | 2 | 0 | 2 | 2 | 0 | | - | - | - | - | - | 12 | |
| Soter et al., 1980 (66) | 2 | 2 | 2 | 2 | 0 | 2 | 2 | 0 | | - | - | - | - | - | 12 | |
| Davis et al., 1981 (67) | 2 | 2 | 2 | 2 | 0 | 2 | 2 | 0 | | - | - | - | - | - | 12 | |
| Kaplan et al., 1981 (68) | 2 | 2 | 2 | 2 | 0 | 2 | 2 | 0 | | - | - | - | - | - | 12 | |
| Lewis et al., 1981 (70) | 2 | 2 | 2 | 2 | 0 | 2 | 2 | 0 | | - | - | - | - | - | 12 | |
| Lawrence et al., 1981 (69) | 2 | 2 | 2 | 2 | 0 | 2 | 2 | 0 | | - | - | - | - | - | 12 | |
| Shelly et al., 1983 (156) | 2 | 2 | 2 | 2 | 0 | 2 | 2 | 0 | | - | - | - | - | - | 12 | |
| Mayou et al., 1986 (157) | 2 | 2 | 2 | 2 | 0 | 2 | 2 | 0 | | - | - | - | - | - | 12 | |
| Adachi et al., 1994 (158) | 2 | 2 | 2 | 2 | 0 | 2 | 2 | 0 | | - | - | - | - | - | 12 | |
| Kato et al., 1997 (11) | 1 | 2 | 2 | 2 | 0 | 2 | 2 | 0 | | - | - | - | - | - | 11 | |
| Fukunaga et al., 2005 (71) | 2 | 2 | 2 | 2 | 0 | 2 | 2 | 0 | | - | - | - | - | - | 12 | |
| Takahagi et al., 2009 (72) | 2 | 2 | 2 | 2 | 0 | 2 | 2 | 0 | | 2 | 2 | 2 | 0 | 2 | 20 | |
| Kozaru et al., 2011 (76) | 2 | 2 | 2 | 2 | 0 | 2 | 2 | 0 | | - | - | - | - | - | 12 | |
| Kim et al., 2015 (74) | 2 | 2 | 2 | 2 | 0 | 2 | 2 | 0 | | 2 | 2 | 0 | 0 | 2 | 18 | |
| Washio et al., 2017 (73) | 2 | 2 | 2 | 2 | 0 | 0 | 2 | 0 | | - | - | - | - | - | 10 | |
| Iijima et al., 2017 (75) | 2 | 2 | 2 | 2 | 0 | 2 | 2 | 0 | | - | - | - | - | - | 12 | |
| Nishioka et al., 1984 (85) | 2 | 2 | 2 | 2 | 0 | 2 | 2 | 0 | | - | - | - | - | - | 12 | |
| Carrillo et al., 1986 (78) | 2 | 2 | 2 | 2 | 0 | 2 | 2 | 0 | | - | - | - | - | - | 12 | |
| Turjanmaa et al., 1989 (79) | 2 | 2 | 2 | 2 | 0 | 2 | 2 | 0 | | - | - | - | - | - | 12 | |
| Fernández de Corres et al., 1993 (80) | 2 | 2 | 2 | 2 | 0 | 2 | 2 | 0 | | - | - | - | - | - | 12 | |
| Quirce et al., 1993 (81) | 2 | 2 | 2 | 2 | 0 | 2 | 2 | 0 | | - | - | - | - | - | 12 | |
| Lezaun et al., 1994 (82) | 2 | 2 | 2 | 2 | 0 | 2 | 2 | 0 | | - | - | - | - | - | 12 | |
| Yamakawa et al., 2001 (83) | 2 | 2 | 2 | 2 | 0 | 2 | 2 | 0 | | - | - | - | - | - | 12 | |
| B. Non-randomized controlled trials | | | | | | | | | | | | | | | |  |
| Study, year (Ref) | **Criteria** | | | | | | | | **Additional criteria in the case of comparative study** | | | | | | | |
|  | **A stated aim of the study** | **Inclusion of consecutive patients** | **Prospective collection of data** | **End point appropriate to the study aim** | **Unbiased evaluation of end points** | **Follow-up period appropriate** | **Loss to follow-up not exceeding 5%** | **Prospective calculation of the study size** | | **A control group having the criterion standard intervention** | **Contemporary groups** | **Baseline equivalence of groups** | **Prospective calculation of the sample size** | **Statistical analyses adapted to the study design** | **Total** | |
| Adachi et al., 2003 (84) | 2 | 2 | 2 | 2 | 0 | 2 | 2 | 0 | | - | - | - | - | - | 12 | |
| Nater et al., 1977 (161) | 2 | 2 | 2 | 2 | 0 | 2 | 2 | 0 | | - | - | - | - | - | 12 | |
| Taskila et al., 2000 (86) | 1 | 2 | 2 | 2 | 0 | 2 | 2 | 0 | | - | - | - | - | - | 11 | |
| Sibbald et al., 1981 (88) | 2 | 2 | 2 | 2 | 0 | 2 | 2 | 0 | | - | - | - | - | - | 12 | |
| Czarnetzki et al., 1986 (90) | 2 | 2 | 2 | 2 | 0 | 2 | 2 | 0 | | - | - | - | - | - | 12 | |
| Gimenez-Arnau et al., 1992 (89) | 2 | 2 | 2 | 2 | 0 | 2 | 2 | 0 | | - | - | - | - | - | 12 | |

-, high risk of bias; 0, not reported; 1, reported but inadequate; 2, reported and adequate.

**Supplementary Table 2** Evidence of histamine release in symptomatic dermographism (SD)

| **Study**  **(year)** | **Number of patients** | **Histamine studies** | | | |
| --- | --- | --- | --- | --- | --- |
|  |  | ***In vitro*** | | ***In vivo*** | |
|  |  | **Method** | **Result** | **Method** | **Result** |
| Rose(20)  (1941) | 10 | - | - | -venous histamine level obtained before, after scratching an area extending from  the nape of the neck to the waist for 5 min  *Measure: Using guinea-pig ilea in a superfusion cascade system*(118, 119)  (normal level: N/A) | - markedly increased histamine level in 2 patients   -baseline: 7.0-8.0 y/100 ml  -peak level: at 5 min (16.0-40.0 y/100 ml)   - slightly increased histamine level in 3 patients   -baseline: 4.0-7.0 y/100 ml  -peak level: at 5-15 min (5.5-10.0 y/100 ml) |
| Greaves(17)  (1970) | 8 | - | - | -histamine levels assessed in subcutaneous perfusate before and after firm stroking of the skin at 15 min  *Measure: Histamine activity was assessed using guinea-pig ilea in 8 patients with SD and 16 control subjects* | rising histamine levels after stroking  histamine activity  Patient 1: at 30-45 min (4.5 ng/ml)  Patient 2: at 15-30 min (3.5 ng/ml)  Patient 3: at 45-60 min (50.0 ng/ml)  Patient 4: at 15-30 min (20.0 ng/ml)  Patient 5: at 75-90 min (8.0 ng/ml)  Patient 6: at 30-45 min (9.0 ng/ml)  Patient 7-8: at 0-15 min (20 ng/ml)  (no histamine was detected in the 16 control subjects) |
| Garafalo(21)  (1981) | 1 | - | - | -venous blood histamine level obtained from antecubital vein before and after scratching circumferentially of the same forearm  *Measure: radioenzyme technique*(98) (normal level: N/A) | rising level  -baseline: 11, 18, 33 ng/ml on 3 separate occasion  -peak level after stimulation: at 2 min (62 ng/ml) |
| Lawlor(120)  (1989) | 6 | - | - | -(I) mast cell number and histamine level in skin biopsy obtained after application of 0.05% clobetasol propionate for 6 weeks compared with application of base (control)  *Measure of histamine: single isotope radioenzyme assay*(100) | -mast cell number and histamine level were markedly decreased after 6 weeks application of 0.05% clobetasol propionate compared with control   - mast cell count:   1.75±1.18 vs 19.5±3.84  -histamine level (ng/mg): 1.1±0.47 vs 4.5±1.7 |

Abbreviations: I, indirect evidence; mg, milligrams; min, minutes; ml, milliliters; N/A, not available/applicable; ng, nanograms; SD, symptomatic dermographism; vs, versus

**Supplementary Table 3** Evidence of histamine release in cold urticaria (ColdU)

| **Study**  **(year)** | **Number of patients** | **Histamine studies** | | | |
| --- | --- | --- | --- | --- | --- |
|  |  | ***In vitro*** | | ***In vivo*** | |
|  |  | **Method** | **Result** | **Method** | **Result** |
| Rose(20)  (1941) | 2 | - | - | -venous histamine level obtained before, after immersion of arm in 5^o^C water for 5 min  *Measure: Using guinea-pig ilea in a superfusion cascade system*(118, 119)  (normal level: N/A) | rising level  Patient 1: baseline: 3.0 y/100 ml  peak level: at 15 min  (14.0 y/100 ml)  Patient 2: baseline: 4.5 y/100 ml  peak level: at 15 min  (6.0 y/100 ml) |
| Henderson(121)  (1958)^†^ | 1 | - | - | 1.venous histamine level obtained before, after immersion in water at 10^o^C for 7 min (three occasions)  2.urine histamine collected during the 11 hours prior to the test and 2 hours after test  *Measure: Assay on an isolated strip of guinea pig ileum*(122)  (normal level: N/A) | 1.blood histamine: rising level   - whole blood histamine   -baseline: 1.6-2.3 mcg/100 ml  -at 5-6 min: 22.0-47.6 mcg/100 ml   - plasma histamine   -baseline: 0.4-1.5 mcg/100 ml  -at 5-6 min: 33.0-70.0 mcg/100 ml  2.urine histamine  -before challenge: 0.68 mcg/hr  -after challenge: 1.31 mcg/hr |
| Dunér(25)  (1960) | 4 | - | - | -venous histamine level obtained before, during, after immersion of hand and forearm in 6-7^o^C water for 5-10 min compared with 14 healthy controls  *Measure: Using isolated guinea-pig*  *ileum in a 3 ml bath containing Tyrode's solution at 38^o^C*  (normal level: N/A) | rising level in both patients and controls  Patients: baseline: normal limit  (5.3-19.8 mcg/100 ml)  after challenge: rising level in 3  patients (12.1-22.3 mcg/100 ml)  Controls: baseline: normal limit  after challenge: rising level in 13  patients |
|  | 1 | - | - | -venous histamine level obtained before and after  1) exposure of right hand to cold with application of tourniquet at right upper arm (venous stasis)  2) contralateral site | 1) challenged site  -baseline: 8.5 mcg/100 ml  -after 5 min exposure: 12.2 mcg/100 ml  2) contralateral site  -baseline: 8.2 mcg/100 ml  -after 5 min exposure: 8.8 mcg/100 ml |
| Juhlin(34)  (1961) | 3 |  |  | -(I) skin biopsy from patients  Patient 1: the first section from normal skin and the second section from urticarial lesion after challenge  Patient 2, 3: skin biopsy was obtained   1. before ice contact 2. after ice contact for 15 min   after ice contact for 15 min and then rewarming for 5 min | -Patient 1: no different in both sections  -Patient 2, 3: only patient 2’s specimen after ice contact for 15 min showed markedly depleted ghostlike or degranulated mast cells. |

| **Study**  **(year)** | **Number of patients** | **Histamine studies** | | | |
| --- | --- | --- | --- | --- | --- |
|  |  | ***In vitro*** | | ***In vivo*** | |
|  |  | **Method** | **Result** | **Method** | **Result** |
| Spuzic(26)  (1961) | 7 | - | - | -plasma histamine level before and after washing with cold water (10^o^C) until the appearance of the symptoms (~3-5 min)  *Measure: biological assay*(118)  (normal level: N/A) | rising plasma histamine level in 3 patients  Patient 2: baseline: 36 mcg/l  10-12 min: 107 mcg/l  Patient 5: baseline: 43 mcg/l  10-12 min: 58 mcg/l  Patient 6: baseline: 61 mcg/l  10-12 min: 1,028 mcg/l |
| Beall(123)  (1963) | 1 | - | - | -plasma histamine concentration before and after immersion of forearm to an ice bath at 12^o^C for 5 minutes  *Measure: fluorescence assay* (normal level: 10 μg/l) | 1.rising plasma histamine level after challenge  -baseline: 3.1 mcg/l  -after 5 min exposure: 13.0 μg/l  2.after 36 mg chlorpheniramine daily ingestion for 6 weeks  -baseline: 5.7 mcg/l  -after left arm cool 6^o^C: the symptoms were delayed in appearance and much less severity, but the levels were greatly elevated.   - 5 min: 28.0 mcg/l - 10 min: 20.4 mcg/l |
| Granerus(124)  (1969) | 1 | - | - | -urine histamine, methylhistamine, and methyl imidazole acetic acid level obtained during seven consecutive days  -this patient had cold urticaria at 4^th^-5^th^ day from taking baths and at 6^th^ day from riding bicycle  *Measure: N/A* | -striking increased in urinary excretion of histamine and its metabolites during days when the patient had attack of cold urticaria |
| Kaplan(18)  (1975)^‡^ | 6 | - | - | -venous blood histamine level before and after immersion of hand into a bucket of ice (0^o^C) for 4 min (various intervals up to 20 min)  *Measure: radioenzyme technique*(98) (normal level: N/A) | 1.rising level in all 5 patients without systemic symptoms  -baseline: ̴ 0 ng/ml  -peak level: at 4-6 min (ranging from 10-36 ng/ml)  2.rising level in a patient with systemic symptoms  -baseline: ̴ 0 ng/ml  -peak level: at 4 min (260 ng/ml) |
| Kaplan(31)  (1976)^‡^ |  |  |  |  |  |
| Soter(27)  (1976) | 3 | - | - | -venous blood histamine level obtained from antecubital fossa before and after immersion in ice water for 3 min (various intervals until 1 hour)  *Measure: radioenzyme technique*(98) (normal level: 0.1 ng/ml) | rising level in all patients  -baseline: 0 mg/ml  -peak level: at 5 min (ranging from 15-70 ng/ml)  -returned to baseline by 30 min |
| Bentley-Phillips(28)  (1976) | 5 | - | - | -venous blood histamine level obtained before and after immersion in water 15^o^C for 5 min compared with after cold tolerance treatment  *Measure: Using guinea-pig ilea in a superfusion cascade system*(96, 97)  (normal level: 2.5 ng/ml) | rising level  -baseline: N/A  -level at 2 min after termination of immersion in water 15^o^C for 5 min: 10-20 ng/ml  -level after cold tolerance treatment: 0 ng/ml |
|  |  |  |  |  | *(continued)* |

| **Study**  **(year)** | **Number of patients** | **Histamine studies** | | | |
| --- | --- | --- | --- | --- | --- |
|  |  | ***In vitro*** | | ***In vivo*** | |
|  |  | **Method** | **Result** | **Method** | **Result** |
| Bentley-Phillips(125)  (1978) | 13 | - | - | -venous blood histamine level obtained before and after immersion in cold water for 5 min compared with after treatment with doxantrazole  *Measure: Using guinea-pig ilea in a superfusion cascade system*(96, 97)  (normal level: 2.5 ng/ml) | 1.rising level before treatment  -baseline: 0 ng/ml  -peak level: at 5 min (14.8 ± 1.9 ng/ml)  2.reduced level after treatment  -baseline: 0 ng/ml  -peak level: at 5 min (8.2 ± 2.2 ng/ml) |
| Kaplan(3)  (1978) | 5 | - | - | -histamine level in blister fluid from applying suction blister over urticarial skin (after ice cube challenge) and contralateral normal skin  *Measure: radioenzyme technique*(98) (normal level: N/A) | rising level at affected site; 13.6-127.7 ng/ml  (control site: 3.0-16.3 ng/ml) |
| Black(30)  (1980) | 6 | - | - | -venous blood histamine level obtained before and after immersion of forearms in 10^o^C water for 5 min (various intervals) compared between before and after being received prednisolone)  *Measure: Using guinea-pig ilea in a superfusion cascade system*(96, 97)  (normal level: 1-2.5 ng/ml) | 1.rising level after cold challenge in 6 patients  -baseline: 0 ng/ml  -peak level: at 2-7 min (ranging from 4.4-51 ng/ml)  2.reduced level after taking prednisolone and then cold challenge in 5 patients  -baseline: 0 ng/ml  -peak level: at 2-7 min (ranging from 0-17.6 ng/ml) |
| Black(126)  (1979) | 12 | - | - | -venous blood histamine level obtained before and after cold challenge compared between before and after cold tolerance induction  *Measure: N/A* (normal level: N/A) | 1.before induction  -peak level: within 10 min (10-20 ng/ml)  -returned to baseline levels within 30 min  2.after induction  -little or no rise in histamine level |
| Sigler(29)  (1979)^§^ | 1 | - | - | -venous blood histamine level obtained before and after immersion of hand in ice water for 4 min (various intervals up to 30 min)  *Measure: radioenzyme technique*(98) (normal level: N/A) | rising level (detected histamine level at 2 min after challenge)  -baseline: 0 ng/ml  -peak level: at 8 min (28 ng/ml)  -returned to baseline by 20 min |
| Inoue(127)  (1980) | 1 | - | - | -venous blood histamine level obtained before and after immersion of forearm in 15^o^C water bath for 5 min (various intervals)  *Measure: spectrofluorometry*(128)  (normal level: N/A) | rising level  -baseline: ̴ 9.2 ng/ml  -peak level: at 10 min ( ̴ 23.3 ng/ml; 2-fold from baseline)  -returned to baseline at 15 min |
|  |  |  |  |  | *(continued)* |

| **Study**  **(year)** | **Number of patients** | **Histamine studies** | | | |
| --- | --- | --- | --- | --- | --- |
|  |  | ***In vitro*** | | ***In vivo*** | |
|  |  | **Method** | **Result** | **Method** | **Result** |
| Keahey(129)  (1980) | 6 | - | - | -venous blood histamine level obtained before and after immersion of hand and forearm in 8-10^o^C cold water for 5 min (various intervals) comparing between before and after being received aminophylline and albuterol  *Measure: Using guinea-pig ilea in a superfusion cascade system*(96, 97)  (normal level: 1 ng/ml) | 1.rising level  -baseline: ̴ 11.14 ng/ml  -mean peak level: at 5 min (20.2 ± 4.7 ng/ml)  2.the level was lower after being received aminophylline and albuterol  -baseline: ̴ 10.29 ng/ml  -mean peak level: at 5 min  (13.2 ± 3.7 ng/ml) |
|  | 3 | - | - | -venous blood histamine level obtained before and after immersion of hand and forearm in 8-10^o^C cold water for 5 min (various intervals) comparing between before and after being received prednisolone  *Measure: Using guinea-pig ilea in a superfusion cascade system*(96, 97)  (normal level: 1 ng/ml) | Patient 1:   - Before treatment:   -peak level: time N/A  (14.0 ± 2.5 ng/ml)   - After treatment: undetectable   Patient 2:   - Before treatment:   -peak level: 7 min (19.0 ng/ml)   - After treatment: 1 ng/ml   Patient 3:   - Before treatment:   -peak level: 7 min (11.0 ng/ml)   - After treatment: 20.0 ng/ml |
| Sigler(36)  (1980) | 6 | - | - | -venous blood histamine level obtained before and after immersion of hand in ice-water bath for 5 min (various intervals) compared between before and after being received cyproheptadine  *Measure: radioenzyme technique*(98) (normal level: N/A) | 1.rising level  -baseline: 0-2.6 ng/ml  -peak level: at 2-6 min (ranging from 5.7-83 ng/ml)  2.no difference in the magnitude of histamine release in 5 patients before or after therapy.  -baseline: 0-8.6 ng/ml  -peak level: at 0-9 min (ranging from 7-65 ng/ml) |
| Akiyama(130)  (1981) | 1 | - | - | -venous blood histamine level obtained before and after immersion of hand into a bucket of ice water for 3 min (various intervals up to 25 min)  *Measure: fluorometric method*(128)  (normal level: N/A) | rising level  -baseline: 0 ng/ml  -peak level: at 5 min ( ̴ 126.3 ng/ml)  (controls had no rising histamine level; about 5 ng/ml) |
|  |  | - | - | -(I) skin biopsy obtained  1) the site where the ice cube was never done  2) from an urticarial lesion induced 15 min after the ice cube test  3) from the skin where the ice cube test was repeated 12 times over 2 days and biopsied 1 hour after the final one | -skin biopsy showed degranulation of the mast cells in specimen 3 |
|  |  |  |  |  | *(continued)* |

| **Study**  **(year)** | **Number of patients** | **Histamine studies** | | | |
| --- | --- | --- | --- | --- | --- |
|  |  | ***In vitro*** | | ***In vivo*** | |
|  |  | **Method** | **Result** | **Method** | **Result** |
| Black(131)  (1981) | 6 | - | - | -venous blood histamine level obtained before and after immersion of forearm in cold water at 10^o^C for 5 min (various intervals up to 30 min) compared with after taking 20-mg prednisolone  *Measure: Using guinea-pig ilea in a superfusion cascade system*(96, 97)  (normal level: N/A) | 1.rising level  -baseline: ̴ 1.2 ng/ml  -peak level: at 2-7 min (4.4-51.0 ng/ml)  2.level was lower after treatment  -baseline: 0 ng/ml  -peak level: at 2-15 min (1.0-20.0 ng/ml) |
| Kaplan(35)  (1981) | 11 | -(I) skin biopsy was done and divided into 4 fragments  1: maintained at 37^o^C for 45 min  2: frozen at 4^o^C for 45 min  3: frozen at 4^o^C for 15 min and then warmed at 37^o^C for 30 min  4: boiled for 30 min  *Measure: radioenzyme technique*(98) | rising histamine level in skin biopsy which frozen at 4^o^C for 15 min and then warmed at 37^o^C for 30 min (fragment 3) | - | - |
| Kaplan(132)  (1981)^¶^ | 4 | - | - | -venous blood histamine level obtained before and after sitting at cold room at 4^o^C for 10-20 min (various intervals)  *Measure: radioenzyme technique*(98) (normal level: N/A) | rising level in 3 patients  -baseline: < 2 ng/ml  -peak level: at 15-20 min (ranging from 4-6 ng/ml) |
| Johnston(133)  (1982)^ω^ | 1 | - | - | -venous histamine level obtained before, during, and after placing hand in ice water for 4 min  -arterial histamine level obtained at before induction of anesthesia, before and after aortic cross-clamping (the patient was cooled to 31^o^C), and during warming in cardiopulmonary bypass procedure^ω^  *Measure: N/A* (normal level: N/A) | 1.venous histamine level obtained from cold challenge  -baseline: ̴ 700 pg/ml  -peak level: at ̴ 8 min ( ̴ 2,500 pg/ml; 4-fold increase above baseline)  2.arterial histamine level obtained in bypass procedure  -normal level before anesthesia  -rising level after anesthesia with peak level during rewarming from 31^o^C to 38^o^C |
| Wasserman(134)  (1982) | 8 | - | - | -venous blood histamine level obtained before and after immersion of arm into ice water for 3 min | rising level  -baseline: 1.1 ± 0.4 ng/ml  -peak level: at 5 min (40 ± 15 ng/ml)  -returned to baseline level by 30 min |
| Katayama(135)  (1983) | 3 | - | - | -venous blood histamine level obtained before and after immersion of hand into ice water for 4 min (various intervals)  *Measure: spectrofluorometry*(136) (normal level: 0.5 ng/ml) | rising level  Patient 1: baseline 10 pmol/ml  peat level: at 5 min  (52 pmol/ml)  Patient 2: baseline 50 pmol/ml  peat level: at 5 min  (97 pmol/ml)  Patient 3: baseline 4 pmol/ml  peat level: at 5 min  (60 pmol/ml) |
|  |  |  |  |  | *(continued)* |

| **Study**  **(year)** | **Number of patients** | **Histamine studies** | | | |
| --- | --- | --- | --- | --- | --- |
|  |  | ***In vitro*** | | ***In vivo*** | |
|  |  | **Method** | **Result** | **Method** | **Result** |
| Kaplan(137)  (1984)ǂ | 1  cold-dependent dermato-  graphism | - | - | -venous blood histamine level obtained before and after being scratch circumferentially at forearm and sitting at cold room for 18 min (various intervals)  *Measure: radioenzyme technique*(98) (normal level: 100 pg/ml) | rising level  -baseline: < 100 pg/ml  -peak level: at 19 min (3,100 pg/ml) |
|  | 1  systemic cold urticaria | - | - | -venous blood histamine level obtained before and after sitting at cold room for 25 min (every 5-10 min intervals)  *Measure: radioenzyme technique*(98) (normal level: 100 pg/ml) | rising level  -baseline: 0 pg/ml  -peak level: at 20 min (5,000 pg/ml) |
| Neittaanmäki(138)  (1984) | 7 | - | - | -histamine level in blister fluid from applying suction blister over skin which provoked by two consecutive cold challenge for 20 min each, compared with controls  *Measure: single-isotope radioenzyme assay* | -histamine concentration in patients’ blisters were higher than control blister  -peak histamine concentration in patients’ blister fluid was at 5 min ranging from 6.0-51.2 ng/ml, while, control blisters showed minor fluctuation levels during post-challenge period of 30 min |
| Wasserman(139)  (1984) | 5 | - | - | -venous blood histamine level obtained before and after immersion of arm in an ice water bath for 3 min  *Measure: single-isotope radioenzyme assay*(140) (normal level: 0.5 ng/ml) | rising level  -baseline: N/A, but elevation was observed until 5 min (22.8 ng/ml)  -peak level: at 10 min (29.8 ng/ml)  -fell to 2.8 ng/ml at 20 min |
| Grandel(112)  (1985) | 6 | - | - | -venous blood histamine level obtained before and after immersion of forearm in ice water at 4-8^o^C for 3 min *Measure: sensitive single-isotope radioenzyme assay*(140)  (normal level: 0.2 ng/ml) | rising level  -baseline: 0 pg/ml  -peak level: time N/A  (< 0.2 - 22.4 ng/ml) |
| Farnam(48)  (1986)ǂǂ | 1 | - | - | -venous blood histamine level obtained before and after staying in a 4^o^C room for 15 min compared with treatment with cimetidine four times per day  *Measure: radioenzymatic procedure*(99) (normal level: 263 ± 202 pg/ml) | 1.rising level  -baseline: ̴ 169.2 pg/ml  -peak level: at 20 min  (600 pg/ml; 3-fold over baseline)  2.after treatment with cimetidine  -baseline: 250 pg/ml  -peak level: at 10 min (> 1,000 pg/ml)  -declined at 20 min |
| Heavey(111)  (1986) | 4 | - | - | -venous blood histamine level obtained before and after immersion of forearm in 10^o^C water for 5 min  *Measure: double isotope radio-enzymatic method)*(141) (normal level: N/A) | rising level  -baseline: 0.24 ± 0.09 ng/ml  -peak level: at 6 min  (ranging from 16.9-96.9 ng/ml) |
| Gruber(8)  (1988) | 1 | -(I) basophil histamine release after incubation with a cold urticaria serum with high titer IgM anti-IgE antibodies  *Measure: radioenzyme technique*(98) (normal level: 100 pg/ml) | -%histamine release was 28.6.  -significant decreased of histamine release when incubation with IgE-sepharose or anti-IgM immunoabsorbant | - | - |
|  |  |  |  |  | *(continued)* |

| **Study**  **(year)** | **Number of patients** | **Histamine studies** | | | |
| --- | --- | --- | --- | --- | --- |
|  |  | ***In vitro*** | | ***In vivo*** | |
|  |  | **Method** | **Result** | **Method** | **Result** |
| Keahey(142)  (1988) | 1 | - | - | -venous blood histamine level  1.obtained before and after immersion of forearm in cold water bath (8-10^o^C) for 2.5 min (various intervals up to 50 min)  2.compared with induction of tolerance (repetitive exposure of hand and forearm in ice water bath)  *Measure: modified microenzymatic assay*(140) (normal level: N/A) | 1.rising level  -baseline: 178 ± 35 pg/ml (n=2)  -peak level: at 6 min (53,154 pg/ml)  -returned to baseline during the ensuing 44 min  2.level before induction of tolerance was higher  -baseline: N/A  -peak level: at 4 min (15,784 pg/ml)  -returned to baseline during the ensuing 16 min |
|  | 1 | - | - | -(I) electron microscopy of skin biopsy obtained  1.before cold challenge  2.20 min after cold-induced angioedema  3.20 min after challenge and induction of tolerance | numerous mast cells undergoing exocytotic change in specimen of post-challenge (specimen 2) |
| Ormerod(110)  (1988) | 6 | - | - | -venous blood histamine level obtained before and after immersion of arm in water at 10^o^C for 5 min (various intervals)  *Measure: radioenzyme technique*(100) (normal level: N/A) | rising level  -baseline: < 0.5 ng/ml  -peak level: at 10 min ( ̴ 11.3 ng/ml) |
| Maltby(113)  (1989) | 5 | - | - | -venous blood histamine level obtained before and after immersion of one hand into ice/water at 0^o^C for 3 min (various intervals)  *Measure: double isotope radio-enzymatic method*(141) (normal level: N/A) | rising level in 3 patients  Patient 1: baseline 0.31 ng/ml  peak level 4.79 ng/ml  Patient 2: baseline 1.11 ng/ml  peak level 3.56 ng/ml  Patient 3: baseline 0.59 ng/ml  peak level 2.22 ng/ml |
| McClean(14)  (1989) | 1 | - | - | -venous blood histamine level obtained before and after staying in cold room at 6^o^C for 15 min (various intervals)  *Measure: radioenzymatic assay technique*(99) (normal level: 400 ± 250 pg/ml) | rising level  -baseline: 357 pg/ml  -peak level: time N/A (556 pg/ml) |
| Anfosso-Capra(143)  (1990) | 1 | - | - | -venous blood histamine level obtained before and after immersion of forearm for 4 min in 4^o^C cold water in patient received second venom desensitization for 1 year  *Measure: sensitive radioimmunoassay*  (normal level: N/A) | rising level  -baseline: 0.4 ng/ml  -peak level: at 1 min (1.4 ng/ml) |
| Orfan(144)  (1991) | 1 | - | - | -venous blood histamine level obtained shortly after placing ice cube test on forearm for 5 min  -patient was place in a 6^o^C room and then serum was obtained for histamine evaluation  *Measure: radioimmunoassay kit*  (normal level: N/A) | rising level  -baseline: 0.9 ng/ml (ice cube test was completed just before baseline)  -peak level: at 20 min in cold room (2 ng/ml)  -returned to normal within 10 min after removal from the cold room |
|  |  |  |  |  | *(continued)* |

| **Study**  **(year)** | **Number of patients** | **Histamine studies** | | | |
| --- | --- | --- | --- | --- | --- |
|  |  | ***In vitro*** | | ***In vivo*** | |
|  |  | **Method** | **Result** | **Method** | **Result** |
| Miller(145)  (1992) | 1 | - | - | -venous blood histamine level obtained at  1) baseline  2) after local cold stimulation test (ice cube test) from challenged site  3) after generalized cold exposure (sitting in a cold room at 4^o^C) from contralateral site  -compared with normal subjects  *Measure: fluorometry*  (normal level: 4-14 mcg/dl) | 1.local cold stimulation test:  rising level compared with baseline  2.generalized cold exposure:  rising level  (normal controls: normal level throughout testing procedure) |
| Rosenkranz(146)  (1992) | 8 | - | - | -venous blood histamine level obtained before and after immersion of hand in water at 4-8 ^o^C (various intervals)  *Measure: radioimmunoassay-kit (Immunotech)* (normal level: < 1 ng/ml) | rising level in 6 patients  -baseline: 1 ng/ml  -peak level: at 10 min (15 ng/ml) |
| Tillie-Leblond(108)  (1994)ǂǂǂ | 5  One patient was treated with cimetidine (400 mg/day) and ketotifen (1 mg/day) for 6 months | - | - | -venous blood histamine level obtained before and after immersion of the right hand into chilled water (4^o^C) for 4 min (various intervals)  -one patient was performed again after treatment.  *Measure: competitive radioimmunoassay (Immunotech)* (normal level: N/A) | 1.rising level within the first minute after challenge in 3 patients who developed urticaria (other 2 patients did not develop any local reaction)  -baseline: N/A  -peak level of 3 patients: 64, 55, 38 nmol/L  2.when compared with other 2 patients with no symptoms  -peak level of these 2 patients: 6.6, 5.8 nmol/L  3.when compared with the level measured after treatment  -peak level: 4 nmol/L  (before treatment: 64 nmol/L) |
|  | 4 | - | - | -(I) electron microscopy of skin biopsy at 10 min after the ice-cube test  -one patient was performed again after treatment. | -the electron microscopy showed many degranulated mast cells in the pericapillary zone.  -In 1 patient who underwent biopsy after treatment: intact granules were detected in mast cells |
| Andersson(32)  (1995) | 3 | - | - | -microdialysis method to determine histamine level at baseline and after ice challenge  *Measure: radioimmunoassay (Immunotech) in the skin microdialysis* (normal level: 0.5 nM) | rising level  -baseline: 6.7-15.0 nM  -peak level: at 6-26 min (91.0-549.9 nM)  (peak level of controls: 33.4-41.1 nM) |
| Capulong(147)  (1997) | 1 | - | - | -venous blood histamine level obtained before and after immersion of left forearm in a basin with ice water at 4^o^C for 10 min compared with opposite site (various intervals; expressed in percentage based on the histamine content)  *Measure: HPLG* (normal level: N/A) | rising level   - 1^st^ challenge: opposite site   -baseline: ̴ 57.3% histamine release  -peak level: at 15 min  (68.2% histamine release)   - 2^nd^ challenge: challenged site   -baseline: ̴ 51.8% histamine release  -peak level: at 15 min  (59.1% histamine release) |
|  |  |  |  |  | *(continued)* |

| **Study**  **(year)** | | **Number of patients** | | **Histamine studies** | | | | | | | |
| --- | --- | --- | --- | --- | --- | --- | --- | --- | --- | --- | --- |
|  |  |  |  | ***In vitro*** | | | | ***In vivo*** | | | |
|  |  |  |  | **Method** | | **Result** | | **Method** | | **Result** | |
| Asero(148)  (2002) | 5 | | -(I) sera from affected patients were tested basophil histamine release assays (basophils were taken from 6 donors)  *Measure: N/A* (normal level: 5% HRA) | | -histamine release was detected in all patients’ tests from basophils of 2/4 donors  -at follow-up visit: histamine release was detected only in patient 3 who was clinical remission. (patient 1 (clinical remission) and 2 (active disease) were not detected histamine) | | - | | - | |  |
| Nuutinen(33)  (2007) | | 6 | | - | | - | | -microdialysis method to determine histamine concentration at baseline and after ice cube challenge  *Measure: radioenzyme assay in the skin microdialysis* (normal level: 0.5-1.0 nmol/l) | | rising level  -baseline: < detected limit  -peak level: at 15-30 min (347 ± 507 nM)  -histamine concentration was declined in the subsequent 15-min fractions for up to 2 hours | |
| Ota(149)  (2010) | | 1 | | - | | - | | -venous histamine level obtained before and 5 min after placing left hand on ice  *Measure: N/A* (normal level: N/A) | | rising level  -baseline: 0.76 ng/ml  -peak level: at 5 min (216 ng/ml) | |
| Kring Tannert(10)  (2012) | | 7 | | - | | - | | -microdialysis method to determine histamine concentration at baseline and after cold exposure before desensitization and after desensitization  *Measure: fluorescence spectroscopy in the skin microdialysis* (normal level: N/A) | | 1.rising level  -baseline: N/A  -peak level after cold exposure but before desensitization 8 min  (149 ± 50 nM; n=6)  -peak level after cold exposure and desensitization (49 ± 34 nM; n=4)  2.In 3 patients, histamine level was rising in 8 min after the exposure but before desensitization (140 nM).  -subsequent stimulation with codeine gave rise to low amounts of histamine release (35 nM ).  -after desensitization, the histamine was rising low level (41 nM) after cold exposure, whereas the subsequent  codeine stimulation resulted in  higher amount of histamine release. | |
| Ellis(150)  (2013)^ω^ | | 1 | | - | | - | | -serum histamine level obtained at before bypass procedure, at the onset of cardiopulmonary bypass, once the patient was cooled to 28^o^C, after completion of circulatory arrest, once rewarming was completed, and at 2, 4, and 6 hours after rewarming^ω^  *Measure: N/A* (normal level: N/A) | | rising serum histamine level at onset of bypass (when patients was cooled to 28 ^o^C)  -baseline: 7 nmol/l  -peak level: 8 nmol/l | |
|  | |  | |  | |  | |  | | *(continued)* | |

| **Study**  **(year)** | **Number of patients** | **Histamine studies** | | | |
| --- | --- | --- | --- | --- | --- |
|  |  | ***In vitro*** | | ***In vivo*** | |
|  |  | **Method** | **Result** | **Method** | **Result** |
| Krause(12)  (2013) | 20 | - | - | -microdialysis method to determine histamine concentration at before and after cold provocation (the patients were ramdomized to be received placebo, 20-mg bilastine, and 80-mg bilastine)  *Measure: Histareader^TM^ (REFlab, Copenhagen, Denmark)*  (normal level: N/A) | At 0-20 min dialysis   - untreated patients   -baseline: 19.2 ± 1.8 ng/ml  -after provocation: 108.0 ± 18.1 ng/ml   - patients treated with 20-mg bilastine   -baseline: N/A  -after provocation: 5.7-fold from baseline   - patients treated with 80-mg bilastine   -baseline: N/A  -after provocation: 6.4-fold from baseline  At 20-60 min dialysis   - untreated patients   -baseline: 20.9 ± 2.1 ng/ml  -after provocation: 35.7 ± 6.2 ng/ml   - patients treated with 20-mg bilastine   -baseline: N/A  -after provocation: 23.7 ± 3.2 ng/ml   - patients treated with 80-mg bilastine   -baseline: N/A  -after provocation: 23.5 ± 3.1 ng/ml |
| Meyer(101)  (2013) | 7 | - | - | -venous histamine level obtained before and after immersion of hand in 10^o^C water for 5 min  *Measure: competitive enzyme immunoassay*  (normal level: N/A) | rising level  -baseline: N/A  -peak level: at 5 min of rewarming (45.5 nM)  -when they compared histamine level at baseline and 10 min following cold challenge at the peak of vascular response.  -histamine concentration at 10 min was significantly increased in patients, while controls were unchanged.  -serum histamine increased the most in the patients with high severity of physical urticaria. |
|  |  | - | - | -(I) skin biopsy obtained at baseline and 15 min after cold stimulation time testing and then stained with tryptase to evaluate mast cell degranulation | -mast cells showed increase of tryptase staining in surrounding tissue and numerous extracellular mast cell  granules after cold challenge |
|  |  |  |  |  | *(continued)* |

| **Study**  **(year)** | **Number of patients** | **Histamine studies** | | | |
| --- | --- | --- | --- | --- | --- |
|  |  | ***In vitro*** | | ***In vivo*** | |
|  |  | **Method** | **Result** | **Method** | **Result** |
| Gorczyza(151)  (2019) | 19 | - | - | -(I) intracutaneous injection with 0.05 ml of histamine, codeine and saline after being received rupatadine 20 mg or placebo daily for 1 week each with randomization  (2-week washout period between treatments)  -wheal and flare diameters were measured with 15 minutes thereafter  -cold provocation testing with Temp*Test* 3.0  Was used to measure CTT in each patient before and after treatment | -in rupatadine 20 mg-received group, wheal sizes were smaller in histamine- and codeine-induced and CTT reduced when compared with placebo.  -the diameters of histamine-induced wheals were 29% smaller |

^†^The patient had both cold urticaria and heat urticaria and was diagnosed with cryoglobulinemia.(121)

^‡^Kaplan et al. (1975) and Kaplan et al. (1976) reported the same patients.(18, 31)

^§^The patient had cold urticaria and cholinergic urticaria.(29)

^¶^The study reported 4 patients with cold-induced cholinergic urticaria.(132)

ǂThe study reported 1 patient with cold-dependent dermatographism and another one with systemic cold urticaria.(137)

ǂǂThe patient reported had both heat-induced and cold-induced urticaria.(48)

ǂǂǂOf the 5 patients in this study, 3 had concomitant exercise-induced urticaria and dermographism.(108)

^ω^The patients in the study of Johnston et al. and Ellis et al. were subjected to circulatory arrest for bypass procedure treatment of coronary artery disease. The patients received a combination of systemic corticosteroid and antihistamine at pre-operative preparation, before bypass, and post-operation.

Abbreviations: CTT, critical temperature threshold; dl, deciliters; HRA, histamine release assay; I, indirect evidence; Ig, immunoglobulin; l, liters; mg, milligrams; mcg, micrograms; min, minutes; ml, milliliters; N/A, not available/applicable; ng, nanograms; nmol, nanomoles; pg, picograms; pmol, picomoles

**Supplementary Table 4** Evidence of histamine release in delayed pressure urticaria (DPU)

| **Study**  **(year)** | **Number of patients** | **Histamine studies** | | | |
| --- | --- | --- | --- | --- | --- |
|  |  | ***In vitro*** | | ***In vivo*** | |
|  |  | **Method** | **Result** | **Method** | **Result** |
| Kaplan(3)  (1978) | 1 | - | - | -histamine level assessed in suction blister fluid from urticarial skin induced by applying 20-lb weight for 10 min (every 2-hour intervals) compared with contralateral site  *Measure: radioenzyme technique*(98)  (normal level: N/A) | -rising level at tested site between 4-8 hours  -baseline: ̴ 4.5 ng/ml  -peak level: at 6-8 hours ( ̴ 31.5 ng/ml) |
| Czarnetzki(38)  (1984) | 7 | - total cellular histamine and %histamine release, on stimulation with ionophore A 23187, from patients’ leukocytes compared with control subjects  *Measure: fluorimetric method*(128)  (normal level: N/A) | -reduced total cellular histamine in patients  (patients vs control subjects:  75.8 ± 29.9 vs 163.5 ± 135.6 ng/2 x 10^6^ cells)  -enhanced % histamine release in patients  (patients vs control subjects:  32.3 ± 10.0 vs 21.7 ± 6.4) | -skin tests were performed by using intradermal injection of 0.01% histamine, 0.01% compound 48/80, 0.01% concanavalin A, and saline into the back.  -pressure was applied using 900 and 1800 g weights with a special device for 10 and 20 min over the back  -skin test sites were examined immediately after removal of the weights and for up to 20 hr.  -histamine level in suction blister fluid from wheals, control sites (untreated patients’ skin) and skin of control subjects  *Measure: fluorimetric method*(128)  (normal level: N/A) | - no significant differences in histamine levels in suction blister fluid between: pressure-, concanavalin A- and compound 48/80-induced wheals and control subjects and control sites (untreated patients’ skin). |
| Mijailović(39)  (1997) | 1  (bullous DPU with a history of recurrent anaphylaxis after pressure) | - | - | -venous blood histamine level before and after applying a 10-kg weight with a belt 2 cm in width hung over the volar site of his left forearm for 10 min  *Measure: N/A* | -rising post-provocation histamine level at the tested site  -baseline: 0.42 mmol/l  -14 hours after testing   - left cubital vein: 0.57 mmol/l - right cubital vein: 0.47 mmol/l |

Abbreviations: cm, centimeters; DPU, delayed pressure urticaria; g, grams; I, indirect evidence; kg, kilograms; l, liters; min, minutes; ml, milliliters; mmol, millimoles; N/A, not available/applicable; ng, nanograms; vs, versus

**Supplementary Table 5** Evidence of histamine release in solar urticaria (SolU)

| **Study**  **(year)** | **Number of patients** | **Histamine studies** | | | |
| --- | --- | --- | --- | --- | --- |
|  |  | ***In vitro*** | | ***In vivo*** | |
|  |  | **Method** | **Result** | **Method** | **Result** |
| Soter(41)  (1979) | 2 | - | - | -venous blood histamine level  Patient 1: sensitive to UVB and UVA  Patient 2: sensitive to UVA and visible light  before and after challenge by exposing one arm to UVB (290-320 nm), UVA (320-400 nm), or visible light (400-760 nm) at various intervals (0.5 to 60 min after challenge) compared with unexposed site  *Measure: radioenzyme assay* | 1.rising level at urticarial lesion of challenged site  -baseline: <0.1 ng/ml  -peak level: at 5 min (7.0 and 37.0 ng/ml)  -fell to baseline levels by 20 min  2.no change of histamine release in unchallenged arms and challenged arms with insufficient intensity of UVA |
| Baart de la Faille(44)  (1975) | 1 | - | - | -(I) skin biopsy from back and dorsal aspect of the forearm before and 24-hour after 3 MED of 403 nm, and from unirradiated skin (thighs and buttocks)  -stained tissue by toluidine blue or the naphthol AS-D chloro-acetate esterase procedure | -mast cells appeared more numerous than normal and tissue from 24-hour after irradiation seemed to have degranulation. |
| Kaplan(3)  (1978) | 1 | - | - | -histamine level in blister fluid from applying suction blister over urticarial skin (after challenge with UV light for 30 sec) and contralateral normal skin  *Measure: radioenzyme technique*(98) (normal level: N/A) | -rising level at affected site: 127 ng/ml  (control site: 24 ng/ml) |
| Hawk(42)  (1980) | 4 | - | - | -venous blood histamine level obtained before and after UVB irradiation for 2, 5, 10, 40 min  -venous blood histamine level obtained at opposite non-irradiated arm in 2 patients  *Measure: Using guinea-pig ilea in a superfusion cascade system*(96, 97) (normal level: N/A) | Challenged forearm  -rising level in 2/4 patients after irradiation  Patient 1: baseline: < 1 ng/ml  peak level: at 2 min (20 ng/ml)  Patient 2: baseline: < 1 ng/ml  peak level: at 5 min (8.5 ng/ml)  (Patient 3 and 4 showed smaller increases)  Contralateral forearm showed  Patient 1: 4.5 ng/ml  Patient 3: 2.5 ng/ml |
|  | 4 | - | - | -(I) electron microscopy of skin biopsy obtained after challenge | -numerous mast cells undergoing exocytotic change |
| Keahey(6)  (1984) | 3 | - | - | -venous blood histamine level obtained before and after UVA irradiation (various intervals up to 30 min) compared with after induction of tolerance (repeated exposure to UVA)  *Measure: microenzymatic method*(152) (normal level: 5 ng/ml) | 1.rising level after irradiation (pre-tolerance)  Patient 1: baseline: 3.9 ng/ml  peak level: at 15 min (7.0 ng/ml)  Patient 2: baseline: 4.3 ng/ml  peak level: at 6 min (11.8 ng/ml)  Patient 3: baseline: 4.3 ng/ml  peak level: at 12 min (6.1 ng/ml)  2.histamine level obtained after induction of tolerance was lower  Patient 1: baseline: 0 ng/ml  peak level: at 12 min (2.4 ng/ml)  Patient 2: baseline: 4.4 ng/ml  peak level: at 15 min (6.5 ng/ml)  Patient 3: baseline: 3.2 ng/ml  peak level: at 6 min (3.9 ng/ml) |

| **Study**  **(year)** | **Number of patients** | **Histamine studies** | | | |
| --- | --- | --- | --- | --- | --- |
|  |  | ***In vitro*** | | ***In vivo*** | |
|  |  | **Method** | **Result** | **Method** | **Result** |
| Keahey(6)  (1984) | 3 | - | - | -(I) electron microscopy of skin biopsy obtained in 3 conditions  1: before challenge  2: 15 min after challenge  3: 15 min after challenge and induction of tolerance | ultrastructural change in mast cells during granulation in tissue from after challenge (condition 2) |
| Neittaanmäki(153)  (1989) | 1 | - | - | 1.histamine level in blister fluid from applying suction blister over urticarial skin (before and after challenge with UVA for 5 min) and contralateral normal skin  2.venous blood histamine level obtained before and after UVA irradiation for 5 min  *Measure: radioenzyme technique*(154) (normal level: N/A) | 1.histamine level in blister fluid  -baseline: N/A  -peak level: at ̴ 5 min (550 nM)  2.plasma histamine level  -baseline: N/A  -peak level: at ̴ 12-13 min (50 nM) |
| Watanabe(43)  (1999) | 2 | - | - | -venous histamine level obtained during slide projector exposure for 15 min and subsequent inhibition spectrum (Y-52) irradiation for 10 min to the forearm  *Measure: RIA (BML, Tokyo, Japan)* | -plasma histamine levels were not elevated during exposure to slide projector light and the subsequent Y-52 filter irradiation. (initial level: <2 ng/ml)  -histamine levels were markedly elevated when  wheals developed after the termination of Y-52 filter irradiation. (level: >10 ng/ml 10 min after termination of irradiation) |

Abbreviations: I, indirect evidence; MED; minimal erythema dose; min, minutes; ml, milliliters; N/A, not available/applicable; ng, nanograms; UV, ultraviolet; UVA, ultraviolet A; UVB, ultraviolet B

**Supplementary Table 6** Evidence of histamine release in heat urticaria (HeatU)

| **Study**  **(year)** | **Number of patients** | **Histamine studies** | | | |
| --- | --- | --- | --- | --- | --- |
|  |  | ***In vitro*** | | ***In vivo*** | |
|  |  | **Method** | **Result** | **Method** | **Result** |
| Rose(20)  (1941) | 1 | **-** | **-** | -venous histamine level obtained before, after immersion of forearm in 48^o^C water for 5 min  *Measure: Using guinea-pig ilea in a superfusion cascade system*(118, 119)  (normal level: N/A) | rising level  -baseline: 6.0 y/100 ml  -peak level: at 5 min (9.0 y/100 ml)  (The patient developed syncope, hypotension, and involuntary micturition and defecation.) |
| Atkins(51)  (1981) | 1 | **-** | **-** | -venous blood histamine level obtained before and after immersion of hand in water at 39-40^o^C for 5 min  (various intervals)  *Measure: radioenzyme technique*(152) (normal level: N/A) | rising level  -baseline: 2-5 ng/ml  -peak level: within 5 min (22.18-135 ng/ml) |
| Grant(52)  (1981) | 1 | **-** | **-** | -venous blood histamine level obtained before and after immersion of right forearm in water bath at 44^o^C for 4 min (various intervals)  *Measure: radioenzyme technique*(98) (normal level: N/A) | rising level   - 1^st^ time of challenging   -baseline: <1 ng/ml  -peak level: at 9 min (7 ng/ml)  -level fell after 9 min but remained rising for 24-min period of sampling   - 2^nd^ time of challenging   -baseline: N/A  -peak level: at 9 min (5 ng/ml)  -level gradually fell over the following 30 min |
| Irwin(49)  (1985) | 2 |  |  | -venous blood histamine level obtained before and after immersion of wrist in water at 56°C for 5 min (various intervals)  *Measure: radioenzyme assay* (normal level: 200-300 pg/ml) | rising level  -baseline: <200pg/ml  -peak level: patient 1 10 min > 3000 pg/ml and patient 2 at 15 min > 2000 pg/ml |
| Koro(53)  (1986) | 1 |  |  | -venous blood histamine level obtained before and after immersion of the elbow at 44.5°C for 5 min.  *Measure: cascade superfusion bioassay* | rising level  -baseline: not detectable, < 2.5 ng/ml  -peak level: 12.5 ng/ml at 8 min |
| Farnam(48)  (1986)* | 1 | - | - | -venous blood histamine level obtained before and after immersion of knees in a tub filled with hot water adjusted to 44^o^C for 20 min compared with treatment with doxepin 75 mg two times per day  *Measure: radioenzymatic procedure*(99)  (normal level: 263 ± 202 pg/ml) | 1.rising level  -baseline: 300 pg/ml  -peak level: at 30 min (800 pg/ml)  2.after treatment with doxepin  -baseline: ̴ 83.3 pg/ml  -peak level: at 30 min  (566.7 pg/ml; 6-fold from baseline)  -declined at 40 min |

| **Study**  **(year)** | **Number of patients** | **Histamine studies** | | | |
| --- | --- | --- | --- | --- | --- |
|  |  | ***In vitro*** | | ***In vivo*** | |
|  |  | **Method** | **Result** | **Method** | **Result** |
| Neittaanmäki(15)  (1988)^†^ | 2 | **-** | **-** | -histamine level in blister fluid from applying suction blister over normal skin after heat challenge at 45^o^C for 7 min (various intervals)  *Measure: radioenzyme assay*(155) (normal level: N/A) | rising level  -baseline: ̴ 1.2 ng/ml  - peak level: at 30 min  ( ̴ 34.7 ng/ml; 29 times compared with baseline) |
| McClean(14)  (1989) | 1 | - | - | -venous blood histamine level obtained before and after immersion of legs in 44^o^C water for 20 min (various intervals)  *Measure: radioenzymatic assay technique*(99) (normal level: 400 ± 250 pg/ml) | rising level  -baseline: 592 pg/ml  -peak level: time N/A (2,181 pg/ml) |
| Higgins(54)  (1991) | 1 | **-** | **-** | -venous blood histamine level obtained before and after immersion of hand in water at 45°C for 5 min (various intervals)  *Measure: radioimmunoassay* | rising level  -baseline: 0.2-0.3 ng/ml  -peak level: at 5 min (12.3 ng/ml) |
| Baba(50)  (1998) | 1 | **-** | **-** | -venous blood histamine level obtained before and 10 min after immersion of hands in hot water at 42^o^C  *Measure: radioimmunoassay*(98)  (normal level: 0.11-0.5 ng/ml) | rising level  -baseline: 0.26 ng/ml  -after testing: 7.64 ng/ml |
| Skrebova(55)  (2001) | 1 | **-** | **-** | -venous blood histamine level obtained before and after immersion of hand in hot water at 43^o^C for 5 min  *Measure: N/A* (normal level: 0.11-0.5 ng/ml) | rising level  -baseline: 0.71 ng/ml  -after testing: 16.6 ng/ml |
| Fukunaga(56)  (2002) | 1 | - | - | -venous blood histamine level obtained before and 10 min after immersion of hand and forearm in water at 40^o^C for 15 min  *Measure: radioimmunoassay*  (normal level: N/A) | rising level after heat challenge about 10 times compared with pre-challenge  (level: N/A) |
| Koh(58)  (2002) | 1 |  |  | -(I) electron microscopy of skin biopsy at the involved wheals and of uninvolved areas obtained at 30 min after heat exposure | -electron microscopy showed features of mast cell degranulation |
| Martín-Muñoz(57)  (2002) | 1 | - | - | -venous blood histamine level obtained before and after immersion of right hand in hot water at 40^o^C (various intervals) compared to non-challenged site  *Measure: competitive enzyme immunoassay methods (Immunotech)* (normal level: N/A) | rising level  -baseline:   - right arm: 3.52 nmol/l - left arm: 3.62 nmol/l   -peak level:   - right arm: at 3 min (8.33 nmol/l) - left arm: at 3 min   (5.46 nmol/l) |

^†^The patient reported had both heat urticaria and cold urticaria.(15)

Abbreviations: I, indirect evidence; l, liters; min, minutes; ml, milliliters; N/A, not available/applicable; ng, nanograms; nmol, nanomoles; pg, picograms

**Supplementary Table 7** Evidence of histamine release in vibratory angioedema (VA)

| **Study**  **(year)** | **Number of patients** | **Histamine studies** | | | |
| --- | --- | --- | --- | --- | --- |
|  |  | ***In vitro*** | | ***In vivo*** | |
|  |  | **Method** | **Result** | **Method** | **Result** |
| Hereditary vibratory angioedema | | | | | |
| Metzger(60)  (1976)^†^ | 1 | - | - | -venous blood histamine level obtained before and after vibratory stimulation by application of forearm on vortex mixer for 4 min (various intervals)  *Measure: radioimmunoassay*(98) (normal level: N/A) | rising level after stimulation  -baseline: 0 ng/ml   - 1^st^ time of challenging   -peak level: at ̴ 3 min (22.4 ng/ml)   - 2^nd^ time of challenging   -peak level: at ̴ 1 min (53 ng/ml) |
| Kaplan(31)  (1976)^†^ |  |  |  |  |  |
| Boyden(61)  (2016) | 2 | **-** | **-** | -venous blood histamine level obtained before and after vibratory stimulation by application of forearm on vortex mixer for 4 min  *Measure: competitive enzyme immunoassay* (normal level: N/A) | rising level  -baseline: < 10 nmol/L  -peak level: ̴ 2 min (90-130 nmol/l) |
|  | 1 | **-** | **-** | -(I) immunohistochemical staining of tryptase in skin sample obtained before and after challenge | The granular contents of mast cells after vibration were released widespread in a patient sample and limited in a control sample. |
| Acquired vibratory angioedema | | | | | |
| Ting(62)  (1983) | 1 | - | - | -venous blood histamine level obtained before and after vibratory stimulation for 1 min (various intervals)  *Measure: fluorometric method*(103)  (normal level: N/A) | rising level  -baseline: 0 ng/ml  -peak level: at 1 min  ( ̴ 24.2 ng/ml) |
|  | 1 | - | - | -(I) electron microscopy of skin biopsy obtained after challenge | mast cells in stimulated site were in various stages of degranulation. |
| Wener(63)  (1983) | 1 | - | - | -venous blood histamine level obtained before and after vibratory stimulation (various intervals)  *Measure: radioenzyme assay*(106) (normal level: < 1 ng/ml) | rising level  -baseline: ̴ 1.5 ng/ml  -peak level: at 5 min (18 ng/ml) |

| **Study**  **(year)** | **Number of patients** | **Histamine studies** | | | |
| --- | --- | --- | --- | --- | --- |
|  |  | ***In vitro*** | | ***In vivo*** | |
|  |  | **Method** | **Result** | **Method** | **Result** |
| Keahey(5)  (1987) | 2 | - | - | -venous blood histamine level obtained before and after vibratory stimulation for 5 min (various intervals)  *Measure: modified microenzymatic assay*(140) (normal level: N/A) | rising level after stimulation  Patient 1: baseline: N/A  peak level: at 5 min  (4,500-9,000 pg/ml)  Patient 2: baseline: 200 pg/ml  peak level: at 5 min  (1,224 pg/ml)  *These 2 patients had rising histamine again at 3-4 hours |
|  | 2 | - | - | -(I) electron microscopy of skin biopsy obtained after challenge | prominent mast cell degranulation in challenged site |
| Zhao(64)  (2019) | 3 | **-** | **-** | -venous blood histamine level obtained before and after vibratory stimulation  *Measure: N/A* | rising level  -baseline: ̴ 3.46 ng/ml  -peak level: at 30 min  ( ̴ 8.54 ng/ml) |

^†^It should be noted that the study of Metzger et al.(1976) and Kaplan et al.(1976) were reported the same patient. (31, 60)

Abbreviations: I, indirect evidence; l, liters; min, minutes; ml, milliliters; N/A, not available/applicable; ng, nanograms; nmol, nanomoles; pg, picograms; VA, vibratory angioedema

**Supplementary Table 8** Evidence of histamine release in cholinergic urticaria (CholU)

| **Study**  **(year)** | **Number of patients** | **Histamine studies** | | | |
| --- | --- | --- | --- | --- | --- |
|  |  | ***In vitro*** | | ***In vivo*** | |
|  |  | **Method** | **Result** | **Method** | **Result** |
| Kaplan(18)  (1975)^†^  and  Kaplan(31)  (1976)^†^ | 3 | - | - | -venous blood histamine level  before and during running every 5 min intervals to maximum of 35 min  (exercise test for 10 min)  *Measure: radioenzyme technique*(98) (normal level: N/A) | rising plasma histamine level in 2/3 patients  *patient 1*:  - baseline: ̴ 1 ng/ml  - peak level: 8 min (25 ng/ml)  - at 35 min: 4 ng/ml  (not returned to baseline)  *patient 2*:  - baseline: 0.8 ng/ml  - peak level: time N/A (3 ng/ml)  - returned to baseline by 20 min |
|  | 3  added in Kaplan(31)  (1976) | - | - | -venous blood histamine level  before and during running every 5 min intervals to maximum of 35 min  (exercise test for 10 min)  *Measure: radioenzyme technique*(98) (normal level: N/A) | rising plasma histamine level in 1/3 patients  - baseline: < 1 ng/ml  - peak level: time N/A (4 ng/ml) |
| Sigler(29)  (1979)^‡^ | 1 | - | - | -venous blood histamine level  before and during running every 5 min intervals to maximum of 60 min  (exercise test for 20 min)  *Measure: radioenzyme technique*(98) (normal level: N/A) | rising plasma histamine level  - baseline: ̴ 0.34 ng/ml  - peak level: 30 min post-exercise  (18 ng/ml)  (normal level during exercise  rising level at 5 min post-exercise  and remained elevated for >40 min  post-exercise) |
| Soter(66)  (1980) | 7 | - | - | -venous blood histamine level  before, during and after exercise running in various intervals up to 40 min  *Measure: radioenzyme technique*(98) (normal level: ≤ 0.1 ng/ml) | rising plasma histamine level in 7/7 patients  - baseline: 0.6±0.4 ng/ml  - peak level: 20 min (6.2±2.0 ng/ml)  - returned to baseline by 40 min |
| Davis(67)  (1981)^§^ | 1 | -(I) basophil histamine release test  (from patient’s blood)  *Measure: triplicate by the enzymatic double-isotopic assay*(106)  (normal level: N/A) | total cellular histamine  - mean baseline: 63.8 ng/ml  *1^st^ time of challenge*  - nadir level at 30 min:  40.2 ng/ml  *2^nd^ time of challenge*  - peak level at 40 min:  50.9 ng/ml | -venous blood histamine level  before and after immersion in a bathtub of tap water at 41^o^C at 10, 20, 30, 60 and 90 min  (hot bath challenge for 30 min for 2 times)  *Measure: triplicate by the enzymatic double-isotopic assay*(106)  (normal level: ≤ 1 ng/ml) | rising plasma histamine level  *1^st^ time of challenge*  - baseline: < 1 ng/ml  - peak level: 90 min (1.7 ng/ml)  *2^nd^ time of challenge*  - baseline: 1.6 and 1.0 ng/ml  - peak level: 90 min (2.3 ng/ml) |

| **Study**  **(year)** | **Number of patients** | **Histamine studies** | | | |
| --- | --- | --- | --- | --- | --- |
|  |  | ***In vitro*** | | ***In vivo*** | |
|  |  | **Method** | **Result** | **Method** | **Result** |
| Kaplan(68)  (1981) | 2 | - | - | -venous blood histamine level  before and during cycling on stationary bicycle every 10 min intervals to maximum of 60 min  (exercise test for 30 min)  *Measure: radioenzyme technique*(98) (normal level: N/A) | rising plasma histamine level in 2/2 patients  *patient 1*:  - baseline < 2 ng/ml  - peak level: 25 min (8 ng/ml)  *patient 2*:  - baseline < 2 ng/ml  - desensitization with 4 challenges  day 1 - peak level: ̴ 23 min (26 ng/ml)  day 2 - peak level: N/A (14 ng/ml)  day 3 - peak level: N/A (4.5 ng/ml)  day 4 - peak level: N/A (0.8 ng/ml) |
| Lewis(70)  (1981) | 6 | - | - | -venous blood histamine level  before and during running every 5-15 min intervals to maximum of 90 min  *Measure: radioenzyme technique*(98) (normal level: ≤ 2 ng/ml) | rising plasma histamine level in 2/6 patients with systemic symptoms  *patient 1*: baseline ≤ 2 ng/ml  after initiation of exercise  - at 45 min: 11.2 ng/ml (peak level)  - at 55 min: 4.5 ng/ml  - at 80 min: 5 ng/ml  *patient 2*: baseline ≤ 2 ng/ml  after initiation of exercise  - at ̴ 37 min: 23.2 ng/ml (peak level) |
| Lawrence(69) (1981) | 3 |  |  | -venous blood histamine level  before and during running every 5-15 min intervals to maximum of 90 min  Measure: *bio-assay antagonized by mepyramine*  (normal level: ≤ 1 ng/ml) | rising plasma histamine level in 1 patient  - pre-exercise (baseline) = 1 ng/ml  - exercise 10 min  - post-exercise 10 min = 20 ng/ml  (peak level) |
| Shelly(156)  (1983) | 1 | - | - | -(I) electron microscopy of skin biopsy at urticarial wheal | - degranulated mast cells  - swollen endothelial cells in the  smaller vessels  - no increase in the number of  mast cells |
| Mayou(157)  (1986) | 1 | - | - | -(I) electron microscopy of skin biopsy at urticarial wheal  (normal level: 10.1-13.5% of cells degranulating) | - rising percentage of cells  degranulated mast cells  - pre-challenge: 14.7% (mean)  - post-challenge: 31.3% (mean)  - no increase in the number of  mast cells |
|  |  |  |  |  | *(continued)* |

| **Study**  **(year)** | **Number of patients** | **Histamine studies** | | | |
| --- | --- | --- | --- | --- | --- |
|  |  | ***In vitro*** | | ***In vivo*** | |
|  |  | **Method** | **Result** | **Method** | **Result** |
| McClean(14)  (1989) | 4 | - | - | -venous blood histamine level  before and during cycling on stationary bicycle every 5 min intervals to maximum 30 min  (exercise test for 20 min)  *Measure: radioenzymatic assay technique*(99)  (normal level: 0.4 ± 0.25 ng/ml) | rising plasma histamine level in 4/4 patients  *patient 1*:  - baseline < 0.9 ng/ml  - peak level: 10 min (9 ng/ml)  *patient 2*:  - baseline ̴ 0.8 ng/ml  - peak level: 15 min (̴ 5.7 ng/ml)  *patient 3*:  - baseline ̴ 0.4 ng/ml  - peak level: 15 min (̴ 6.3 ng/ml)  *patient 4*:  - baseline ̴ 0.5 ng/ml  - peak level: 25 min (̴ 1.6 ng/ml)  (reduced peak histamine level after taking ketotifen) |
| Adachi(158) (1994) | 5 | -(I) leukocyte histamine release test  of patients’ leukocytes with autologous sweat in 3 concentrations  -(I) leukocyte histamine release test of passive sensitization of leukocytes with serum with autologous sweat in 3 concentrations  *Measure: high-performance liquid chromatography with a cation exchanger combined with an automated Shore’s fluorometric detection system*  (normal level: N/A) | **Normal leukocytes**  the percentage of histamine release was significantly higher than control group  1. unconcentrated sweat  = 33.4%±26.4%  (*P* < 0.05)  2. 10 times concentrated sweat = 62.2%±27.3%  (*P* < 0.01)  3. 50 times concentrated sweat = 77.6%±23.2%  (*P* < 0.01)  **Passive sensitized leukocytes with serum**  1. l/l0 serum dilution  = 58.9% (rising)  2. l/5 serum dilution  = 22.8% (rising)  3. l/2 serum dilution  = 9.5%  4. buffer alone (baseline)  = 10.6% |  |  |
| Kato(11)  (1997) | 1 | - | - | -venous blood histamine level  before and after stepping stair up and down for 10 and 15 min  (exercise test for 5 min)  *Measure: N/A* | rising plasma histamine level  - baseline: 0 ng/ml  - peak level: 15 min (50 ng/ml) |
|  |  |  |  |  | *(continued)* |

| **Study**  **(year)** | **Number of patients** | **Histamine studies** | | | |
| --- | --- | --- | --- | --- | --- |
|  |  | ***In vitro*** | | ***In vivo*** | |
|  |  | **Method** | **Result** | **Method** | **Result** |
| Fukunaga(71)  (2005) | 17 | -(I) basophil histamine release test  incubated patients’ basophils with autologous sweat in various concentrations  *Measure: a commercial kit (Shionogi, Osaka, Japan)*  (normal level: ≤ 5% histamine release) | rising percentage histamine release from patients’ basophils  (1) dilution of autologous sweat 1/100  - 9 patients showed positive response  (ranging from 7.3-100%)  (2) dilution of autologous sweat 1/1000  - 4 patients showed positive response  (ranging from 6.5-36.6%) | - | - |
| Takahagi(72)  (2009) | 35 | -(I) basophil histamine release test  incubated patients’ basophils with semipurified and standardized sweat antigen  *Measure: reversed-phase high performance liquid chromatography*  (normal level: ≤ 5% histamine release) | rising % histamine release from patients’ basophils in 23/35 patients  - baseline: N/A  - peak level: N/A  - mean % of histamine release of patients  = 24.56 ± 4.00 %  - mean % histamine release of controls  = -0.10 ± 0.36 %  (significantly correlated between % histamine with serum total IgE level and numbers of eosinophils) | - | - |
| Kozaru(76)  (2011) | 6 | -(I) histamine release test of sensitized leukocytes by autologous sweat  *Measure: a commercial kit (Shionogi, Osaka, Japan)* | - testing % histamine release in 3/6 patients  **before vs after**  patient 1 = 68.7% vs 20.4%  patient 3 = 33.0% vs 0.0%  patient 4 = 31.0% vs 32.0%  - reducing histamine release  with autologous sweat after rapid desensitization in 2/3 patients |  |  |
| Kim(74)  (2015) | 18 | -(I) basophil histamine release test incubated patients’ basophils with autologous sweat in 1:10 and 1:100 dilutions  *Measure: a histamine EIA kit (Immunotech)*  (normal level: ≤ 5% histamine release) | rising % histamine release from patients’ basophils in 6/18 patients  - baseline: N/A  - peak level: N/A  - mean % of histamine release of patients = ̴ 5.5%  (significantly correlated between % histamine with wheal size in autologous sweat skin test) | - | - |
|  |  |  |  |  | *(continued)* |

| **Study**  **(year)** | **Number of patients** | **Histamine studies** | | | |
| --- | --- | --- | --- | --- | --- |
|  |  | ***In vitro*** | | ***In vivo*** | |
|  |  | **Method** | **Result** | **Method** | **Result** |
| Washio(73)  (2017) | 13 | -(I) histamine release test  against human sweat  *Measure: a commercial kit (Shionogi, Osaka, Japan)* | positive histamine release test 9/13 patients  results: index value  - 1 (weakly positive): 1 patient  - 2 (positive): 0 patient  - 3-4 (strongly positive): 8  patients  * positive correlation between IgE-RAST titers against *Malassezia* species and histamine release with sweat  * negative correlation between IgE-RAST titers against *Candida* species and histamine release with sweat | - | - |
| Iijima(75)  (2017) | 1 | -(I) histamine release test  against human sweat in various concentrations of sweat  *Measure: a commercial kit (Shionogi, Osaka, Japan)* | histamine release ratio  - high ratio at low concentration  - higher ratios at higher concentrations  (class 4 response) | - | - |

^†^It should be noted that the study of Kaplan et al.(1975) and Kaplan et al.(1976) were reported the same patients.(18, 31)

^‡^The study of Sigler et al. studied one patient with cold and cholinergic urticaria.(29)

^§^The patient in the study of Davis et al. had both aquagenic urticaria and cholinergic urticaria.(67)

Abbreviations: CholU, cholinergic urticaria; I, indirect evidence; min, minutes; IgE, immunoglobulin E; N/A, not available/applicable

**Supplementary Table 9** Evidence of histamine release in contact urticaria (ConU)

| **Study**  **(year)** | **Urticariogenic**  **substance** | **Number of patients** | **Histamine studies** | | | |
| --- | --- | --- | --- | --- | --- | --- |
|  |  |  | ***In vitro*** | | ***In vivo*** | |
|  |  |  | **Method** | **Result** | **Method** | **Result** |
| **Immunologic urticaria** | | | | | | |
| Nishioka(85)  (1984) | chlorhexidine | 1 | - | - | -venous blood histamine level  *Measure: fluorometry combined with high-performance liquid chromatography (HPLC)*(136)  (normal level: < 0.1 ng/ml) | rising level  (1) prick test  · before test: 0.35 ng/ml  · 15 min after test: 1.27 ng/ml  * increased > 3-fold level  (2) patch test  · before test: 0.36 ng/ml  · 1 hr after test: 0.61 ng/ml  * increased 2-fold level |
| Carrillo(78)  (1986) | latex | 1 | -(I) leukocyte histamine release  (from patient’s blood)  *Measure: fluorometric assay*(104)  (normal level: ≤ 10%) | positive histamine release with glove antigen and natural latex especially with molecular weight 30 Dalton (level of % histamine release: N/A) | - | - |
| Turjanmaa  (79)  (1989) | latex | 14^†^ | -(I) basophil histamine release test  *Measure: radioimmunoassay*(159)  (normal level: < 20%) | positive histamine release:  13/14 patients (93%)  mean histamine release:  51.5% (range 23.5-92.8%) | -venous blood histamine level  *Measure: N/A*  (normal level: N/A) | mean blood histamine level of all 21 subjects: 49 ng/ml  (range 12-91 ng/ml)  (15 latex allergy subjects and 6 control subjects)  * no difference between patients and controls |
| Fernández de Corres(80)  (1993) | latex  chestnuts  bananas | 6 | -(I) leukocyte histamine release  *Measure: florometric method*(104)  (normal level: <10%) | - positive histamine release for latex 3/6 patients (50%)  - positive histamine release for chestnuts 3/6 patients (50%)  - positive histamine release for bananas 1/6 patients (16.7%) | - | - |
| Quirce(81)  (1993) | *Limonium tataricum* | 1 | -(I) leukocyte histamine release  (from patient’s blood)  *Measure: automated fluorometric method*(104, 105)  (normal level: < 10%) | % histamine release  tested in 4 dilutions  (1:1, 1:10, 1:100, 1:1000)  maximum release: 69%  total release: 103.3 ng/ml | - | - |
| Lezaun(82)  (1994) | cereal flour extracts  - rice  - wheat  - rye  - oats  - barley  - corn  - *Olea europae* | 1 | -(I) leukocyte histamine release (from patient’s blood)  *Measure: automated fluorometric method*(104)  (normal level: < 10%) | % maximum histamine release tested in 4 dilutions (1:5, 1:25, 1:125, 1:625) of the cereal flour extracts and *Olea europae*  - rice 20%  - wheat 14%  - rye 11%  - oats 14%  - barley 13%  - corn 14%  - *O. europae* 25% |  |  |

| **Study**  **(year)** | **Urticariogenic**  **substance** | **Number of patients** | **Histamine studies** | | | |
| --- | --- | --- | --- | --- | --- | --- |
|  |  |  | ***In vitro*** | | ***In vivo*** | |
|  |  |  | **Method** | **Result** | **Method** | **Result** |
| **Immunologic urticaria** | | | | | | |
| Yamakawa(83)  (2001) | rice | 1 | -(I) glass microfiber-based histamine-release test (HRT)  *Measure: HRT kit (Rusika^®^)*(160)  *Grading the results from 0 to 4, according to significant allergen-induced histamine release*  *- grade 4 > cut-off level at 5^-5^*  *lowest allergen concentration*  *- grade 3 > cut-off level at 5^-3^*  *lowest allergen concentration*  *- grade 2 > cut-off level at 5^-2^*  *lowest allergen concentration*  *- grade 1 > cut-off level at 5^-1^*  *lowest allergen concentration*  *- grade 0 under cut-off level*  (normal level: < 15 ng/ml) | grade of HRT kit  - rice allergen: grade 3  - regular rice-washing  water: grade 3  - salt-soluble rice protein:  grade 4  - allergen free technology  rice-washing water:  grade 1  - 16 KDa allergen: grade 2 | - | - |
| Adachi(84)  (2003) | polyvinylpyrrolidone  (PVP, povidone) | 1 | -(I) basophil histamine release test (from patient’s blood)  *Measure: enzyme-linked immunosorbent assay*(107)  (normal level: N/A) | % histamine release  1) 4 sizes of PVP + 1% autologous serum  - PVP-K15  conc 0 mcg/mL 3.8%  conc 0.01 mcg/mL 3.8%  conc 1 mcg/mL 11.4%  - PVP-K30  conc 0 mcg/mL 3.8%  conc 0.01 mcg/mL 8.5%  conc 1 mcg/mL 19.4%  - PVP-K60  conc 0 mcg/mL 3.8%  conc 0.01 mcg/mL 15.4%  conc 1 mcg/mL 28.5%  - PVP-K90  conc 0 mcg/mL 3.8%  conc 0.01 mcg/mL 0%  conc 1 mcg/mL 8.2%  2) 4 sizes of PVP  (PVP-K15, 30, 60, 90)  - none of PVPs induced histamine release without serum | - | - |
|  |  |  |  |  |  | *(continued)* |

| **Study**  **(year)** | **Allergen** | **Number of patients** | **Histamine studies** | | | |
| --- | --- | --- | --- | --- | --- | --- |
|  |  |  | ***In vitro*** | | ***In vivo*** | |
|  |  |  | **Method** | **Result** | **Method** | **Result** |
| **Non-immunologic urticaria** | | | | | | |
| Nater(161)  (1977) | cinnamaldehyde | 1 | -(I) leukocyte histamine release (from patient’s blood)  *Measure: fluorometric assay*(103)  (normal level: N/A) | % histamine release  in each cinnamaldehyde concentration (conc)  (1) *absence* of autologous serum  · 0.3 mg/ml - 7.9, 17.3 %  · 1.0 mg/ml - 54.5, 74.5,  77.1%  · 3.0 mg/ml - 94.4, 98.4%  (2) *presence* of autologous serum  · 0.3 mg/ml - 5.3, 10.6 %  · 1.0 mg/ml - 66.5, 69.2,  70.5 %  · 3.0 mg/ml - 87.8, 94.4 %  * partial suppression by autologous serum in the incubation mixtures | - | - |
| Taskila(86)  (2000) | nettle | 6 | -(I) basophil histamine release tests  *Measure: N/A*  *(normal level: N/A)* | negative (n=3) | - microdialysis method  (histamine probe was inserted intradermally about 5 cm apart in the anterolateral aspect of the brachium)  *(baseline level: 5.1±2.7 nM, n=6)* | - The histamine level  in the 15-min fraction was 100.3 M (range 35±240 nM, n=5), and declined rapidly in the subsequent fractions to 17.3, 11.8, 11.3, 7.8.  - An immediate peak (at 15±45 min) and a weak delayed (at 90±120 min) histamine peak after the nettle challenge were detected with a wide individual variation. |

Abbreviations: HPLC, high-performance liquid chromatography; I, indirect evidence; hr, hour; HRT, histamine release test; mg, milligram; min, minute; ml, milliliters; mm^2^, square millimeter, N/A, not available/applicable; ng, nanograms; PVP, polyvinylpyrrolidone

^†^Total patients with latex allergy were 15 patients in this study but only 14 patients were tested by basophil histamine release.

**Supplementary Table 10** Evidence of histamine release in aquagenic urticaria (AquaU)

| **Study**  **(year)** | **Number of patients** | **Histamine studies** | | | |
| --- | --- | --- | --- | --- | --- |
|  |  | ***In vitro*** | | ***In vivo*** | |
|  |  | **Method** | **Result** | **Method** | **Result** |
| Davis(67)  (1981) | 1 | -(I) basophil histamine release test  (from patient’s blood)  *Measure: triplicate by the enzymatic double-isotopic assay*(106)  (normal level: N/A) | total cellular histamine  - mean baseline: 54.4 ng/ml  (remained within this range throughout the challenge) | -venous blood histamine level  before and after immersion in a bathtub of tap water at 37^o^C (various intervals)  *Measure: triplicate by the enzymatic double-isotopic assay*(106)  (normal level: 1 ng/ml) | rising plasma histamine level  - baseline: < 1 ng/ml  - peak level: 60 min (1.5 ng/ml)  (returned to pre-challenged level at 2 hours) |
| Sibbald(88)  (1981) | 2 | - | - | - venous blood histamine level  *Measure: Using guinea-pig ilea in a superfusion cascade system*(96)  (normal level: N/A) | rising plasma histamine level in 1/2  - baseline: < 1 ng/ml  - after water exposure  · back  6.5 ng/ml  · face  after water exposure 20 minutes  10.5 ng/ml (peak level)  · arm  after acetone and water exposure 5 minutes  17 ng/ml (peak level)  after acetone and water exposure 20 minutes  15 ng/ml |
|  |  | - | - | -(I) skin biopsy at infrascapular region  after immersion in water for 5 min  *Measure: histological microscopic examination*  (normal range of forearm skin: 34-66  mast cells/mm^2^)  (normal percentage of mast cell degranulation: 9-20%) | - number of mast cell density: 62 cells/mm^2^  (normal)  - proportion of mast cell degranulation: 44%  (rising) |
| Czarnetzki(90)  (1986) | 1 | -(I) basophil histamine release test  after incubation of 1x10^7^ peripheral blood leukocytes with several dilutions of human callus extracts  *Measure: spectroflurometric method*(128)  (normal level: N/A) | % histamine release  in 4 callus dilutions  (patient vs control subject)  1:10 – 6.2 vs 2.9  1:100 – 6.2 vs 0.0  1:1,000 – 12.4 vs 0.0  1:10,000 – 3.2 vs 0.0 | - | - |
| Gimenez-Arnau(89)  (1992) | 1 | - | - | -(I) skin biopsy after wheal induction  Measure: histological microscopic examination  (normal: N/A) | - massive degranulation of dermal mast cells |

Abbreviations: AU, aquagenic urticaria; I, indirect evidence; min, minutes; ml, milliliters; mm^2^, square millimeter, N/A, not available/applicable; ng, nanograms

**Supplementary Table 11** The efficacy of antihistamine therapy in each type of chronic inducible urticaria

| **Chronic inducible urticaria** | **AH1** | **fgAH1** | **sgAH1** | **AH2** | **Combination**  **AH1 and AH2** | **The detail of treatment response** |
| --- | --- | --- | --- | --- | --- | --- |
| Symptomatic dermographism  (SD) | effective | variable efficacy | effective | ineffective | variable efficacy | - FgAH1 showed variable responses.(22)  - The superiority of sgAH1 compared with placebo was found for SD.(37)  - Monotherapy with an AH2 was not effective for SD.(22)  - The combination of AH1 and AH2 increased the efficacy of treatment with fgAH1.(22) |
| Cold urticaria  (ColdU) | effective | effective | effective | - | - | - FgAH1 (cyproheptadine) can prevent cold-induced hives.(162)  - The meta-analysis of 9 randomized controlled studies (RCTs) proved sgAH1 efficacy.(163) |
| Delayed pressure urticaria  (DPU) | effective | ineffective | effective | - | ineffective | - FgAH1 (chlorpheniramine and promethazine hydrochloride) was not effective for preventing wheals.(40)  - Two RCT studies investigated in sgAH1 (cetirizine and desloratadine) and revealed the improvement for relieving symptoms.(40)  - The combination of hydroxyzine and cimetidine was not effective for preventing induced DPU lesions in 13 DPU patients.(40) |
| Solar urticaria  (SolU) | effective | - | effective | - | - | - AH1 treatment is of benefit in 35-75% of SolU patients. (45-47)  - SgAH_1_ (fexofenadine) was reported to effectively relieve symptoms (164)  - The patients who treated by the combination of desloratadine and/or fexofenadine and/or cetirizine and montelukast was reported as full remission in 58%-70% and partial remission in 10%. (165, 166) |
| Heat urticaria  (HeatU) | effective | - | effective | - | effective | - Standard dose of sgAH1 have proven effective in 58% patients of all subtypes of HeatU.(59)  - sgAH1 achieved a full symptom relief in 16% patients of all subtypes of HeatU.(59)  - The addition of AH2 obtained a complete or partial resolution in 29% and 71% of patients, respectively.(59) |
| Hereditary vibratory angioedema  (HVA) | ineffective | - | - | - | - | - One case report using AH1 for HVA, with no improvement. |
| Acquired vibratory angioedema  (AVA) | variable efficacy | variable efficacy | variable efficacy | - | variable efficacy | - SgAH1 led to marked, partial, and no improvement in 40%, 40%, and 20%, respectively.(65)  - FgAH1 led to marked, partial, and no improvement1 in 50%, 33.3%, and 16.7%, respectively.(65)  - AH1 (unmentioned name) led to partial improvement and no improvement for one and one patient, respectively.(65)  - The addition of AH2 to the AH1 treatment resulted in partial control with cimetidine but no improvement with ranitidine.(65) |
| Cholinergic urticaria  (CholU) | effective | effective | effective | - | effective | - FgAH1 and sgAH1 are more effective than placebo.(77)  - The combination of AH1 and AH2 was effective in some patients with refractory cases of CholU.(77) |
| Contact urticaria  (ConU) | effective | - | effective | - | - | - SgAH1 are effective in controlling both the number and the duration of wheals in most patients with ConU.(87) |
| Familial aquagenic urticaria (FAquaU) | effective | - | effective | - | - | - Treatment with AH1 showed complete control, marked improvement, and partial improvement in 62.5%, 12.5% and 50%, respectively.(167)  - Treatment with a sgAH1 led to complete control in 83.3% and partial improvement in 16.7%.(167) |
| Acquired aquagenic urticaria  (AAquaU) | variable efficacy | variable efficacy | variable efficacy | - | variable efficacy | - Standard dose of any AH1 led to complete control, marked, partial, and no improvement in 36.7%, 25.6%, 10.3%, and 17.9%, respectively.(167)  - FgAH1 led to complete control, marked, partial, and no improvement in 28.6%, 28.6%, 21.4% and 21.4%, respectively.(167)  - SgAH1 led to complete control, marked, partial, and no improvement in 56%, 12%, 8% and 24%, respectively.(167)  - The combination of AH1 and AH2 showed partial and no improvement in 33.3% and 66.7%, respectively.(167) |

Abbreviation: AH_1_, H1-antihistamines; AH_2_, H2-antihistamines; fgAH_1_, first-generation antihistamines; sgAH_1_, second-generation antihistamines

**Supplementary Table 12** Level of evidence to consider histamine as the mediator in each chronic inducible urticaria

| **Chronic inducible urticaria** | **Details** | **Number of supporting studies** | **Number of opposing studies** | **Levels of evidence** |
| --- | --- | --- | --- | --- |
| **Symptomatic dermographism** | **Total studies**  - Elevation of histamine level in blood | **4**  3 | -  - | **strong** |
|  | - Reduction of mast cell number and histamine activity after clobetasol application | 1 | - |  |
| **Cold urticaria** | **Total studies**  - Elevation of histamine level in blood | **56**  39 | -  - | **strong** |
|  | - Elevation of histamine level in urine | 2 | - |  |
|  | - Elevation of histamine level in blister fluid | 2 | - |  |
|  | - Elevation of histamine level by intradermal microdialysis method | 4 | - |  |
|  | - Detection of mast cell degranulation by skin biopsy | 5 | - |  |
|  | - Reduction of diameters of histamine-induced wheals in rupatadine 20 mg-received group | 1 | - |  |
|  | - Positive leukocyte histamine release test of patients’ blood after challenging | 2 | - |  |
|  | - Elevation of histamine level in skin biopsy | 1 | - |  |
| **Delayed pressure urticaria** | **Total studies**  - Elevation of histamine level in blister fluid | **4**  1 | **1**  1 | **strong** |
|  | - Elevation of histamine level in blood | 1 | - |  |
|  | - Reduction of total cellular histamine | 1 | - |  |
|  | - Elevation of percentage of histamine release | 1 | - |  |
| **Solar urticaria** | **Total studies**  - Elevation of histamine level in blood | **10**  6 | -  - | **strong** |
|  | - Elevation of histamine level in blister fluid | 1 | - |  |
|  | - Detection of mast cell degranulation by skin biopsy | 3 | - |  |
| **Heat urticaria** | **Total studies**  - Elevation of histamine level in blood | **14**  12 | -  - | **strong** |
|  | - Elevation of histamine level in blister fluid | 1 | - |  |
|  | - Detection of mast cell degranulation by skin biopsy | 1 | - |  |
| **Hereditary vibratory angioedema** | **Total studies**  - Elevation of histamine level in blood | **3**  2 | -  - | **strong** |
|  | - Detection of mast cell degranulation by skin biopsy | 1 | - |  |
| **Acquired vibratory angioedema** | **Total studies**  - Elevation of histamine level in blood | **6**  4 | -  - | **strong** |
|  | - Detection of mast cell degranulation by skin biopsy | 2 | - |  |
| **Cholinergic urticaria** | **Total studies**  - Elevation of histamine level in blood | **19**  10 | 1  - | **strong** |
|  | - Detection of mast cell degranulation by skin biopsy | 2 | - |  |
|  | - No elevation of basophil histamine release test from patient’s blood after challenging | - | 1 |  |
|  | - Positive leukocyte histamine release test of patients’ leukocytes after exposing to autologous sweat | 6 | - |  |
|  | - Positive leukocyte histamine release test of patients’ leukocytes after exposing to standardized sweat antigen | 1 | - |  |
| **Immunologic contact urticaria** | **Total studies**  - Elevation of histamine level in blood | **9**  1 | **1**  - | **strong** |
|  | - No change of histamine level in blood between patients and controls | - | 1 |  |
|  | - Positive leukocyte histamine release test of patients’ blood after exposing to antigen | 7 | - |  |
|  | - Positive glass microfiber-based histamine-release test after exposing to antigen | 1 | - |  |
| **Non-immunologic contact urticaria** | **Total studies**  - Elevation of histamine level by intradermal microdialysis method | **2**  1 | **1**  - | **no evidence** |
|  | - Positive leukocyte histamine release test of patients’ blood after exposing to antigen | 1 | 1 |  |
| **Aquagenic urticaria** | **Total studies**  - Elevation of histamine level in blood | **5**  2 | **1**  - | **strong** |
|  | - Detection of mast cell degranulation by skin biopsy | 2 | - |  |
|  | - Positive leukocyte histamine release test of patients’ blood after challenging | 1 | - |  |
|  | - Negative leukocyte histamine release of patients’ blood after exposing to human callus extracts | - | 1 |  |

The Levels of evidence for “association”: *strong*: 3 studies available that find an association in the same direction or ≥ 4 studies available, of which > 66% find a significant association in the same direction and no more than 25% find an opposite association, *weak*: 2 studies available that find a significant association in the same direction or 3 studies available, of which two find a significant association in the same direction and the third study finds no significant association, *no evidence*: ≤ 1 study available, *inconsistent*: remaining cases The levels of evidence for “no association”: *strong*: > 4 studies are available, of which >85% find no significant association, *weak*: > four studies are available, of which >75% find no significant association

**Supplementary Table 13** Future perspectives and possible research areas

| **Topics** |
| --- |
| Why and how trigger factors cause degranulation of mast cells in each subtype of CIndU? |
| The role of cells other than mast cells in the pathogenesis of CIndU |
| Roles of mediators other than histamine that are involved in the induction of signs and symptoms of each CIndU |
| Factors predicting AH_1_ responses in each CIndU |
| The efficacy of H_4_ receptor antagonists in the treatment of patients with CIndU |
| Novel targeted therapies in the treatment of patients with recalcitrant CIndU |
| Biomarkers for treatment responses and prognosis in each CIndU subtype |

Reference

118. Code CF. The Quantitative Estimation of Histamine in the Blood. *J Physiol* (1937) 89(3):257-68. doi: 10.1113/jphysiol.1937.sp003476.

119. Barsoum GS, Gaddum JH. The Pharmacological Estimation of Adenosine and Histamine in Blood. *The Journal of physiology* (1935) 85(1):1-14. doi: 10.1113/jphysiol.1935.sp003298.

120. Lawlor F, Black AK, Murdoch RD, Greaves MW. Symptomatic Dermographism: Wealing, Mast Cells and Histamine Are Decreased in the Skin Following Long-Term Application of a Potent Topical Corticosteroid. *Br J Dermatol* (1989) 121(5):629-34. doi: 10.1111/j.1365-2133.1989.tb08195.x.

121. Henderson LL, Code CF, Roth GM. Increased Blood Histamine in Thermal Intolerance: Report of a Patient with Cryoglobulinemia. *Journal of Allergy* (1958) 29(2):122-9. doi: https://doi.org/10.1016/0021-8707(58)90097-2.

122. Code CF, McIntire FC. Quantitative Determination of Histamine. *Methods Biochem Anal* (1956) 3:49-95. doi: 10.1002/9780470110195.ch3.

123. Beall GN. Plasma Histamine Concentrations in Allergic Diseases. *J Allergy* (1963) 34:8-15. doi: 10.1016/0021-8707(63)90080-7.

124. Granerus G, Svensson SE, Wetterqvist H, White T. The Metabolism of Histamine in a Case of Cold Urticaria. *Acta Allergol* (1969) 24(4):258-60. doi: 10.1111/j.1398-9995.1969.tb03740.x.

125. Bentley-Phillips CB, Eady RA, Greaves MW. Cold Urticaria: Inhibition of Cold-Induced Histamine Release by Doxantrazole. *J Invest Dermatol* (1978) 71(4):266-8. doi: 10.1111/1523-1747.ep12515101.

126. Black AK, Sibbald RG, Greaves MW. Cold Urticaria Treated by Induction of Tolerance. *Lancet* (1979) 2(8149):964. doi: 10.1016/s0140-6736(79)92664-3.

127. Inoue S, Teshima H, Ago Y, Nagata S. Cold Urticaria Associated with Immunoglobulin M Serum Factor. *J Allergy Clin Immunol* (1980) 66(4):299-304. doi: 10.1016/0091-6749(80)90025-1.

128. Shore PA, Burkhalter A, Cohn VH, Jr. A Method for the Fluorometric Assay of Histamine in Tissues. *J Pharmacol Exp Ther* (1959) 127:182-6.

129. Keahey TM, Greaves MW. Cold Urticaria. Dissociation of Cold-Evoked Histamine Release and Urticara Following Cold Challenge. *Arch Dermatol* (1980) 116(2):174-7. doi: 10.1001/archderm.116.2.174.

130. Akiyama T, Ushijima N, Anan S, Takahashi I, Yoshida H. A Case of Cold Urticaria Due to a Serum Factor Belonging to the Ige Class. *J Dermatol* (1981) 8(2):139-43. doi: 10.1111/j.1346-8138.1981.tb02580.x.

131. Black AK, Keahey TM, Eady RA, Greaves MW. Dissociation of Histamine Release and Clinical Improvement Following Treatment of Acquired Cold Urticaria by Prednisone. *Br J Clin Pharmacol* (1981) 12(3):327-31. doi: 10.1111/j.1365-2125.1981.tb01221.x.

132. Kaplan AP, Garofalo J. Identification of a New Physically Induced Urticaria: Cold-Induced Cholinergic Urticaria. *J Allergy Clin Immunol* (1981) 68(6):438-41. doi: 10.1016/s0091-6749(81)90209-8.

133. Johnston WE, Moss J, Philbin DM, Guiney TE, Sisson JH, Buckley MJ, et al. Management of Cold Urticaria During Hypothermic Cardiopulmonary Bypass. *N Engl J Med* (1982) 306(4):219-21. doi: 10.1056/nejm198201283060406.

134. Wasserman SI, Austen KF, Soter NA. The Functional and Physicochemical Characterization of Three Eosinophilotactic Activities Released into the Circulation by Cold Challenge of Patients with Cold Urticaria. *Clinical and experimental immunology* (1982) 47(3):570-8.

135. Katayama I, Doi T, Nishioka K, Maeyama K, Yamatodani A. Acquired Cold Urticaria: H1 and H2 Antagonists Versus Cold Induced Histamine Release. *J Dermatol* (1983) 10(4):377-82. doi: 10.1111/j.1346-8138.1983.tb01152.x.

136. Yamatodani A, Maeyama K, Watanabe T, Wada H, Kitamura Y. Tissue Distribution of Histamine in a Mutant Mouse Deficient in Mast Cells: Clear Evidence for the Presence of Non-Mast-Cell Histamine. *Biochem Pharmacol* (1982) 31(3):305-9. doi: 10.1016/0006-2952(82)90175-7.

137. Kaplan AP. Unusual Cold-Induced Disorders: Cold-Dependent Dermatographism and Systemic Cold Urticaria. *J Allergy Clin Immunol* (1984) 73(4):453-6. doi: 10.1016/0091-6749(84)90354-3.

138. Neittaanmäki H, Karjalainen S, Fräki JE, Kiistala U. Suction Blister Device with Regulation of Temperature: Demonstration of Histamine Release and Temperature Change in Cold Urticaria. *Arch Dermatol Res* (1984) 276(5):317-21. doi: 10.1007/bf00404624.

139. Wasserman SI, Ginsberg MH. Release of Platelet Factor 4 into the Blood after Cold Challenge of Patients with Cold Urticaria. *J Allergy Clin Immunol* (1984) 74(3 Pt 1):275-9. doi: 10.1016/0091-6749(84)90258-6.

140. Dyer J, Warren K, Merlin S, Metcalfe DD, Kaliner M. Measurement of Plasma Histamine: Description of an Improved Method and Normal Values. *J Allergy Clin Immunol* (1982) 70(2):82-7. doi: 10.1016/0091-6749(82)90233-0.

141. Brown MJ, Ind PW, Causon R, Lee TH. A Novel Double-Isotope Technique for the Enzymatic Assay of Plasma Histamine: Application to Estimation of Mast Cell Activation Assessed by Antigen Challenge in Asthmatics. *J Allergy Clin Immunol* (1982) 69(1 Pt 1):20-4. doi: 10.1016/0091-6749(82)90082-3.

142. Keahey TM, Indrisano J, Kaliner MA. A Case Study on the Induction of Clinical Tolerance in Cold Urticaria. *J Allergy Clin Immunol* (1988) 82(2):256-61. doi: 10.1016/0091-6749(88)91008-1.

143. Anfosso-Capra F, Philip-Joet F, Reynaud-Gaubert M, Arnaud A. Occurrence of Cold Urticaria During Venom Desensitization. *Dermatologica* (1990) 180(4):276. doi: 10.1159/000248047.

144. Orfan N, Marhoul J, Lerner C, Lawrence ID. Systemic Cold Urticaria in a Five-Year-Old Boy. *Ann Allergy* (1991) 67(2 Pt 1):143-6.

145. Miller SD, Pritchard D, Crowley JP. Blood Histamine Levels Following Graded Cold Challenge in Atypical Acquired Cold Urticaria. *Ann Allergy* (1992) 68(1):27-9.

146. Rosenkranz AR, Wekkeli M, Hippmann G, Benda H, Jarisch R, Götz M. Cold Urticaria as a Model of Mediator Release: Platelet Factor 4, Eosinophil Cationic Protein and Histamine. *Allergy* (1992) 47(4 Pt 2):366-70. doi: 10.1111/j.1398-9995.1992.tb02073.x.

147. Capulong MC, Tomikawa M, Tahara K, Akasawa A, Iikura Y. Cold Stimulation Test and Histamine Release in Primary Acquired Cold Urticaria. *Int Arch Allergy Immunol* (1997) 114(4):400-3. doi: 10.1159/000237701.

148. Asero R, Tedeschi A, Lorini M. Histamine Release in Idiopathic Cold Urticaria. *Allergy* (2002) 57(12):1211-2. doi: 10.1034/j.1398-9995.2002.23893_3.x.

149. Ota M, Kawasaki H, Horimoto M. Ice Cream Urticaria. *Am J Med* (2010) 123(12):e1-2. doi: 10.1016/j.amjmed.2010.04.034.

150. Ellis AK, Saha T, Arellano R, Zajac A, Payne DM. Successful Management of Cold-Induced Urticaria During Hypothermic Circulatory Arrest. *Ann Thorac Surg* (2013) 96(5):1860-2. doi: 10.1016/j.athoracsur.2013.03.030.

151. Gorczyza M, Curto-Barredo L, Krause K, Church MK, Hawro T, Metz M, et al. H(1)-Antihistamine Inhibition of Histamine- and Codeine-Induced Wheals Does Not Predict Response in Chronic Cold Urticaria. *J Allergy Clin Immunol Pract* (2019) 7(6):2043-4. doi: 10.1016/j.jaip.2019.01.030.

152. Levy DA, Widra M. A Microassay for Studying Allergic Histamine Release from Human Leukocytes Using an Enzymic-Isotopic Assay for Histamine. *J Lab Clin Med* (1973) 81(2):291-7.

153. Neittaanmäki H, Jääskeläinen T, Harvima RJ, Fräki JE. Solar Urticaria: Demonstration of Histamine Release and Effective Treatment with Doxepin. *Photodermatol* (1989) 6(1):52-5.

154. Harvima RJ, Harvima IT, Fräki JE. Optimization of Histamine Radio Enzyme Assay with Purified Histamine-N-Methyltransferase. *Clin Chim Acta* (1988) 171(2-3):247-56. doi: 10.1016/0009-8981(88)90150-7.

155. Shaff RE, Beaven MA. Increased Sensitivity of the Enzymatic Isotopic Assay of Histamine: Measurement of Histamine in Plasma and Serum. *Anal Biochem* (1979) 94(2):425-30. doi: 10.1016/0003-2697(79)90385-3.

156. Shelley WB, Shelley ED, Ho AK. Cholinergic Urticaria: Acetylcholine-Receptor-Dependent Immediate-Type Hypersensitivity Reaction to Copper. *Lancet* (1983) 1(8329):843-6. doi: 10.1016/s0140-6736(83)91386-7.

157. Mayou SC, Black AK, Eady RAJ, Greaves MW. Cholinergic Dermographism. *British Journal of Dermatology* (1986) 115(3):371-7. doi: https://doi.org/10.1111/j.1365-2133.1986.tb05754.x.

158. Adachi J, Aoki T, Yamatodani A. Demonstration of Sweat Allergy in Cholinergic Urticaria. *J Dermatol Sci* (1994) 7(2):142-9. doi: 10.1016/0923-1811(94)90088-4.

159. Morel AM, Anfosso FJ, Delaage MA. Radioimmunoassay for Histamine. Application to Histamine Release. *Journal of Allergy and Clinical Immunology* (1985) 75(1):124. doi: 10.1016/0091-6749(85)90212-X.

160. Yamakoshi M, Fujii Y, Nagai H, Ohyama K. [a Novel Diagnostic Method for Allergy "Lucica Hrt"]. *Jpn J Clin Pathol* (1997) 45(2):163-73.

161. Nater JP, De Jong MC, Baar AJ, Bleumink E. Contact Urticarial Skin Responses to Cinnamaldehyde. *Contact Dermatitis* (1977) 3(3):151-4. doi: 10.1111/j.1600-0536.1977.tb03631.x.

162. Kulthanan K, Hunnangkul S, Tuchinda P, Chularojanamontri L, Weerasubpong P, Subchookul C, et al. Treatments of Cold Urticaria: A systematic Review. *J Allergy Clin Immunol* (2019) 143(4):1311-31. doi: 10.1016/j.jaci.2019.02.005.

163. Maltseva N, Borzova E, Fomina D, Bizjak M, Terhorst-Molawi D, Košnik M, et al. Cold Urticaria - What We Know and What We Do Not Know. *Allergy* (2021) 76(4):1077-94. doi: 10.1111/all.14674.
164. Faurschou A, Wulf HC. Synergistic Effect of Broad-Spectrum Sunscreens and Antihistamines in the Control of Idiopathic Solar Urticaria. *Arch Dermatol* (2008) 144(6):765-9. doi: 10.1001/archderm.144.6.765.

165. Levi A, Enk CD. Treatment of Solar Urticaria Using Antihistamine and Leukotriene Receptor Antagonist Combinations Tailored to Disease Severity. *Photodermatol Photoimmunol Photomed* (2015) 31(6):302-6. doi: 10.1111/phpp.12186.

166. Snast I, Lapidoth M, Uvaidov V, Enk CD, Mazor S, Hodak E, et al. Real-Life Experience in the Treatment of Solar Urticaria: Retrospective Cohort Study. *Clin Exp Dermatol* (2019) 44(5):e164-e70. doi: 10.1111/ced.13960.

167. Rujitharanawong C, Kulthanan K, Tuchinda P, Chularojanamontri L, Metz M, Maurer M. A Systematic Review of Aquagenic Urticaria-Subgroups and Treatment Options. *J Allergy Clin Immunol Pract* (2022) S2213-2198(22)00477-9. doi: 10.1016/j.jaip.2022.04.033.
